# Environmental selection and advective transport shape the distribution of two cyst-forming Acantharia clades in the Canadian Arctic

**DOI:** 10.1093/plankt/fbae051

**Published:** 2024-10-04

**Authors:** Mary Thaler, Aurélie Labarre, Connie Lovejoy

**Affiliations:** Département de Biologie and Institut de Biologie Intégrative et des Systèmes (IBIS), 1045 Avenue de la Medicine, Université Laval, Québec City, Québec, G1V OA6, Canada; Département de Biologie and Institut de Biologie Intégrative et des Systèmes (IBIS), 1045 Avenue de la Medicine, Université Laval, Québec City, Québec, G1V OA6, Canada; Département de Biologie and Institut de Biologie Intégrative et des Systèmes (IBIS), 1045 Avenue de la Medicine, Université Laval, Québec City, Québec, G1V OA6, Canada

**Keywords:** radiolaria, biogeography, Canada Basin, Nares Strait, mixotrophy, advective transport, vertical migration

## Abstract

Anthropogenic induced climate perturbations are seen in changes in oceanic circulation patterns, and Arctic water masses defined by salinity are vulnerable to change. Biogeography of marine microbial eukaryotes is expected to be impacted by changes in local environmental conditions and advective processes, but tracking the extent of plankton distribution requires understanding routes for both active and passive tracers. To identify such tracers, we focused on samples collected in the western (Canada Basin) and eastern (Nares Strait); extremes of the Canadian High Arctic that are connected by an east flowing current north of Canada. Sequencing of the V4 region of 18S rRNA revealed that Acantharia, a taxonomically and functionally diverse group of large planktonic protists, were particularly common. Arctic acantharians in our study were dominated by two clades belonging to cyst-forming groups. The distribution of one clade suggested successful advective transport from the Pacific sourced water in the Beaufort Gyre to southern Nares Strait, with cells transported along the northern shelf of the Canadian Arctic. A second clade appeared to be a resident taxon of the Canada Basin whose distribution correlated to local environmental conditions, and detection in deeper samples would be consistent with swarmer formation enabling reestablishment the following year.

## INTRODUCTION

Planktonic microbes (microbial eukaryotes, bacteria and archaea) are largely responsible for global carbon, nitrogen, silica and sulfate biogeochemical cycles (Manganelli *et al.*, 2009) with microbial eukaryotes being integral to both biogeochemistry and marine trophic webs (Worden *et al.*, 2015). Biogeography of microbial plankton can be influenced by the history of the water mass where they live (Martiny *et al.*, 2006; Agogué *et al.*, 2011), including passive transport by advective processes such as ocean circulation (Hamilton *et al.*, 2008; Wilkins *et al.*, 2013; Richter *et al.*, 2022; Ibarbalz *et al.*, 2023). Microbial eukaryotes in the Arctic are also subject to environmental filtering, and higher populations are seen under favorable conditions that may be either predictable, such as seasonal change (Freyria *et al.*, 2021), or transient (Joli *et al.*, 2018). 

Climate perturbations are projected to impact oceanic circulation patterns on a basin-wide scale (IPCC – Intergovernmental Panel on Climate Change, 2021; Mishonov *et al.*, 2024), and the biogeography of planktonic marine microbes and protists is expected to undergo major changes. Because of Arctic amplification, the Arctic Ocean has already been impacted by anthropogenic induced climate change (Comeau *et al.*, 2011; Flores *et al.*, 2019; Ardyna and Arrigo, 2020), resulting in ongoing sea-ice decline (Cai *et al.*, 2021), atmospheric warming (Carvalho and Wang, 2020), surface freshening (Coupel *et al.*, 2015) and changes to circulation (Timmermans and Marshall, 2020), which will have major repercussions for higher trophic levels which depend on planktonic microbes for food.

As a contribution to three separate opportunistic oceanographic missions in 2012, 2013 and 2014, we collected microbial DNA and RNA from the water column of two widely separated regions of the Canadian High Arctic seas. Our initial 18S rRNA gene amplicon survey detected high relative abundance of rhizarian sequences, which were identified as members of the planktonic taxon Acantharia. This group within the radiolarian clade consists mostly of larger protists with biomineralized skeletons (Suzuki and Not, 2015; Biard, 2022). Acantharia have received little attention in the Arctic; however, our findings suggested they may be an overlooked element of Arctic pelagic food webs. The contrasting conditions and distributions in two distinct regions presented an opportunity to evaluate the relative importance of local environmental conditions versus advective transport in determining their biogeography in the High Arctic, particularly in the context of changing sea-ice conditions. We focused on samples from water column communities in years of low and high ice coverage in the Canada Basin (2012 and 2013, respectively), and in the Arctic outflow gateway of Nares Strait, which is hydrographically connected to the Canada Basin as described below.

**Fig. 1:**
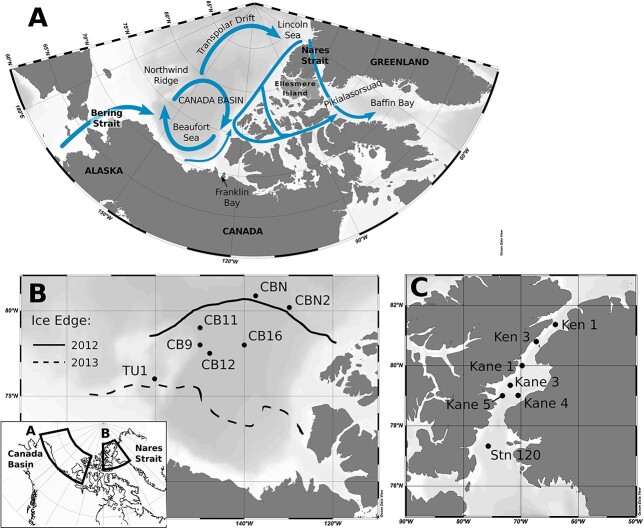
Canadian Arctic sampling stations. (**A**) Schematic showing pathways of Pacific Water circulating through the Canadian Arctic Ocean, based Hu *et al.* (Hu *et al.*, 2019). (**B**) The Canada Basin–Beaufort Sea. Ice extent is traced from charts from the Canadian Ice Services Archive. (**C**) Nares Strait, including northern Baffin Bay (Station 120). Maps were created using Ocean Data View v. 4.7.4 (Schlitzer, 2015).

The Canada Basin, which includes the marginal Beaufort Sea, is characterized by a relatively thick upper layer of fresher water, which results in strong stratification and nearly year-round nutrient limitation in surface waters (McLaughlin and Carmack, 2010). Below this upper layer there is a strong and distinct Pacific Water mass signal originating from waters that have entered the region through Bering Strait (Fig. 1a). This signal is seasonal, with Bering Sea Summer Water (BSSW) being warmer and slightly fresher than Bering Sea Winter Water (BSWW) (Itoh *et al.*, 2012). Nares Strait, on the other side of Canada, flows between Greenland and Ellesmere Island, and is sourced by waters from the Lincoln Sea, which since 1999, is fed by the Beaufort Gyre and contiguous with waters flowing eastward along the northern Canadian continental shelf (Burgers *et al.*, 2017). South-flowing currents leave the Lincoln Sea through Nares Strait, which consists of a series of channels and shallower basins, carrying surface Arctic waters, referred to as Polar Mode Water (PMW); Pacific origin waters (BSSW and BSWW) modified by contact and some mixing with deeper Atlantic Waters, and referred to as Upper Halocline Water (UHW); and deeper Atlantic Water from the Canada Basin (Burgers *et al.*, 2017). Once through the strait, water flows into northern Baffin Bay (*Pikialasorsuaq* or the North Water) (Kalenitchenko *et al.*, 2019).

The large cell size and presence of a cyst-forming life stage in some clades of Acantharia could make them particularly susceptible to large-scale advective transport (Mestre and Höfer, 2020). However, acantharians have historically been underrepresented in the fossil record and microscopy studies on preserved samples, because their celestite (strontium sulfate, SrSO_4_) skeletons dissolve in seawater samples that are undersaturated with strontium (Beers and Stewart, 1970), and they are also absent from sediment samples. Still, their numbers in shallow sediment-traps suggest that Acantharia are responsible for transferring surface ocean carbon out of the euphotic zone in the Southern Ocean (Belcher *et al.*, 2018) and in the East Greenland Sea where Antia *et al.* (Antia *et al.*, 1993) estimated thousands of acantharians per cubic meter. These studies indicate that they reach ecologically-significant abundances in the Polar Oceans including the Arctic. They would contribute to the complexity of food webs as both grazers and prey items and, for a few clades as, mixotrophs with algal symbionts (Decelle *et al.*, 2012a), which likely graze on larger microplankton and nanoplankton similar to the diets of heterotrophic acantharians (Swanberg and Caron, 1991).

Cyst formation is characteristic of the complex life cycles of some Acantharia, with various life stages dominating different depths of the water column (Gilg *et al.*, 2010; Decelle *et al.*, 2013). For example, the acantharians that dominated the East Greenland Sea sediment traps were mostly detected as cysts or as large cells in the process of forming cysts as they absorbed their celestite spicules. The large multi-nucleated vegetative cells are reported from the euphotic zone, where heterotrophs can take advantage of abundant prey, and mixotrophic species with algal symbionts are able to fix carbon in the sunlit surface waters. The large cells can either sink directly or form large cysts that rapidly sink out of surface waters to mesopelagic depths and complete their life cycle by releasing tiny (3–5 μm) single-nucleated swarmer cells, likely a gamete or zygote stage in sexual reproduction (Rizos *et al.*, 2024). At high latitudes, cyst formation appears to be seasonal, with acantharians taking advantage of phytoplankton post-bloom detritus followed by sedimentation to complete their reproductive cycle (Spindler and Beyer, 1990; Martin *et al.*, 2010).

As the form and structure of the skeleton is the primary taxonomic feature of cells (Somu *et al.*, 2023), the identity of the Arctic Acantharia, including cysts from sediment traps, remains speculative. Molecular-based studies are being increasingly used to circumvent methodological difficulties that hinder assessment of acantharian species identity, relative abundance, and diversity (Mars Brisbin *et al.*, 2020), while comparative ribosomal RNA (rRNA) and rRNA gene (rDNA) analyses can provide insight into life stages, with rRNA more likely to represent metabolically active cells, though rRNA copies can also be high in cysts (Blazewicz *et al.*, 2013).

Acantharia detected using 18S rDNA sequences have been reported from water samples in the Canadian Arctic (e.g. Hamilton *et al.*, 2008; Terrado *et al.*, 2009; Lovejoy and Potvin, 2010; Monier *et al.*, 2013; Freyria *et al.*, 2021), but have not been systematically compared across regions. We used amplicon sequence data to explore the distribution of acantharians in two distant regions of the Canadian Arctic. Clades were identified using phylogenetic analysis of the nuclear DNA targeting the complete 18S rRNA gene, or from shorter reads of the gene’s V4 variable region. The rRNA gene products, referred to hereafter as rDNA, tend to be more conserved than rRNA, which can fragment and degrade more easily, though it still provides evidence of living cells with high ribosomal activity. We used statistical analyses to differentiate between the distributions found with rRNA versus rDNA, while ecological context for these distributions was analyzed using rRNA only. Given the oceanographic differences between Canada Basin and Nares Strait, we aimed to determine whether acantharian populations on either side of the Central Arctic were taxonomically related, and if water mass movements could explain their distributions with respect to depth and other variables. Since many protists in the Arctic have pan-Arctic distributions (Thaler and Lovejoy, 2015; Luddington *et al.*, 2016; Dorrell *et al.*, 2022), our null hypothesis was that *in situ* conditions determined species distribution independent of potential source populations.

## METHOD

In the Canada Basin, samples were collected as part of the Joint Ocean Ice Studies—Beaufort Gyre Exploration Project (https://www2.whoi.edu/site/beaufortgyre/) aboard the Canadian Coast Guard ship *CCGS Louis S. St-Laurent* in the Canada Basin and North Wind Ridge area in 2012 and 2013 (Fig. 1b). Water column properties, including salinity, temperature, depth, and *in situ* chlorophyll fluorescence were measured with sensors mounted on the ship’s rosette system (McLaughlin and Carmack, 2010). Briefly, sample depths were chosen based on the downcast profile; for this study we targeted the surface layer, the subsurface chlorophyll maximum (SCM), and the BSWW, which was sampled at around 100 m. Samples were collected during the upcast by closing 10 L Niskin bottles. Six stations were visited from 25–39 August 2012 with 2 to 3 depths per station analyzed. In 2013, five stations were visited from 17–23 August, and 3 depths per station were analyzed (Fig. 1b).

Canada Basin samples for nutrient analyses (nitrate, phosphate, silicate) were collected directly from the Niskin bottles and analyzed using a 3-channel Technicon Auto Analyzer (SEAL Analytical, Mequon WI, USA) following the methods of Barwell-Clarke and Whitney (Barwell-Clarke and Whitney, 1996). Samples for flow cytometry (FCM) were collected following Li and Dickie (Li and Dickie, 2001) and Li *et al.* (Li *et al.*, 2011). Briefly, a 100 mL polycarbonate (PC) bottle was rinsed with sample water and then filled directly from the Niskin bottles. Duplicate 1.8 mL aliquots were immediately transferred to 2.0 mL cryovials and preserved with 200 μL 10% EM grade paraformaldehyde (Sigma-Aldrich). The cryovials were briefly vortexed and left at room temperature in the dark for at least 10 min and then kept at −80°C. Bacteria were stained before analysis with the nucleic acid dye Sybr-Green (Life Technologies, USA). The 2012 samples were analyzed using a FACSort (Becton Dickinson, San Jose, CA) equipped with a 477-nm argon-ion laser. Following cross calibrations, the 2013 samples were processed using an EPICS ALTRA flow cytometer (Beckman Coulter) (Tremblay *et al.*, 2009) and a BD Accuri C6 flow cytometer (BD Biosciences) both equipped with a blue (488 nm) laser.

In northern Baffin Bay and Nares Strait, samples were collected during the 2014 ArcticNet mission aboard the *CCGS Amundsen*. Water column profiling, selection of sample depths, sample collection, and analysis for nutrients and FCM are described in Kalenitchenko *et al.* (Kalenitchenko *et al.*, 2019). Briefly, samples were collected from 4–6 August 2014 with 4 to 8 depths analyzed at the stations indicated in Fig. 1c. Physical and nutrient data for the cruise are available from Amundsen Science (see data availability statement). At each station, samples were taken from near the surface (PMWs), and from the SCM, which corresponded to the location of UHW, consisting of modified Pacific waters (Burgers *et al.*, 2017). To increase the potential water mass coverage, additional depths were collected down the water column. The deepest samples were taken 5–10 m from the bottom, which at most stations corresponded to Canada Basin Atlantic Water (CBAW), depending on the maximum depth of the station.

Ice conditions and extent for both Canada Basin and Nares Strait came from the Canadian Ice Service Archive (Canadian Ice Service, 2007). For all sampling cruises, samples for nucleic acids were collected into acid-cleaned and sample-rinsed carboys and filtered immediately on board. Approximately 6 L of water from each depth was filtered sequentially using a peristaltic pump and an in-line filtration system consisting of a 50 μm mesh to remove macrozooplankton, a 3 μm pore size (large fraction) 47 mm PC filter (AMD Manufacturing) and a 0.2 μm (small fraction) Sterivex filter unit (Millipore). The large fraction filters were placed in 2 mL microfuge tubes. To stabilize the RNA, 1.8 mL of RNALater (Invitrogen, ThermoFisher Scientific) was added to both Sterivex units and the microfuge tubes, which were left at room temperature for 30 min, then stored at −80°C until processing in the laboratory.

DNA and RNA were extracted from the same filter using the All-Prep DNA/RNA Minikit (Qiagen). The RNA was converted to cDNA using the High Capacity Reverse Transcription Kit (Applied Biosystems, ThermoFisher Scientific) following kit instructions. The V4 region of 18S rRNA was amplified from both rDNA and rRNA samples using the forward primer E572F and reverse primer E1009R, each conjugated with a MiSeq specific linking primer (Illumina) as described in Comeau *et al.* (Comeau *et al.*, 2011). Illumina barcodes were added at the 5′ end of both amplicon strands using the TruSeq and Nextera (Illumina) barcode sets in a nested PCR with conditions similar to above except that initial denaturation was for 30 s, subsequent denaturation steps were for 10 s, there were only 13 cycles and the final elongation was 4 min. All amplicons were purified using the Agencourt AMPure XP (Beckman Coulter) magnetic bead method and checked by gel electrophoresis. Concentrations of purified barcoded samples were measured spectrophotometrically with a Nanodrop 2000c UV–Vis (ThermoScientific) and then pooled at a final concentration of 121 ng μL^−1^ per sample before sequencing. Multiplex sequencing was carried out using the MiSeq-Illumina platform at IBIS/Université Laval Plateforme d’Analyses Génomiques (Québec City, Québec, Canada).

### Bioinformatics

Raw read data were quality controlled using FastQC v0.11.9 (Andrews, 2010) (Table S1). Adaptors and low-quality reads were trimmed with AdapterRemoval v2.2.4 (Lindgreen, 2012), with a base quality threshold of 25. Microbial 18S rRNA sequences were filtered with maximum EE scores of 2 and 4 for forward and reverse reads respectively, and trimmed according to the error rate algorithm applied in DADA2 v1.22.0 (Callahan *et al.*, 2016). Subsequently, DADA2 was used to differentiate partial 18S rRNA gene Amplicon Sequence Variants (ASVs). ASVs were assigned a taxonomy using the classification algorithm IDTAXA (Murali *et al.*, 2018) from the Bayesian classifier method implemented in DECIPHER v2.18.1 package (Wright, 2016) using the PR2 database v4.14 (Guillou *et al.*, 2012) as reference. We removed singleton ASVs, as well as ASVs belonging to Bacteria, Metazoa and Streptophyta, to focus on microbial eukaryotes.

To aid the robust identification of Acantharia clades, an 18S rRNA reference tree was constructed using 443 nearly full length sequences of the 18S rRNA gene from the PR2 database (Guillou *et al.*, 2012) and Arctic environmental sequences in Genbank. Sequences were aligned using MAFFT v.7 (Katoh *et al.*, 2005). Alignment sites retained for subsequent phylogenetic analysis were selected using trimAL (Capella-Gutierrez *et al.*, 2009), gap distribution mode and trimming of ambiguously aligned sites was done manually with AliView (Larsson, 2014). Phylogenetic relationships were inferred by the maximum likelihood (ML) approach, model TIM2 + F + R4 and bootstrap methods provided topology support values in IQ-TREE (Nguyen *et al.*, 2015), using 1000 ultrafast bootstrap replicates (Minh *et al.*, 2013).

Phylogenetic analysis was performed using ASVs assigned to two separate clades of Acantharia from rDNA reads only. For Clade B-Northwind Ridge (B-NR), 10 amplicon V4 18S rDNA environmental sequences were aligned with the V4 region from 40 Arctic-specific reference sequences (233 base pair alignment), and for Clade C-Franklin Bay (C-FB), eight amplicon environmental sequences were aligned with the V4 region from 27 reference sequences (240 base pair alignment). Phylogenetic relationships were inferred with topology support values as described above, with the difference that we employed the K3P + R2 model in IQ-TREE (Nguyen *et al.*, 2015). Amplicons with sporadic occurrences and multiple indels in conserved regions were assumed be intragenomic polymorphisms (Weber and Pawlowski, 2014) and were not included in the alignments or trees.

### Statistical analyses

We used a multi-factorial analysis of variance (ANOVA) to explore categorical factors associated with the relative abundance of Acantharia clades, including nucleic acid type (rRNA versus rDNA), size fraction (< 3 μm versus > 3 μm), water mass and sampling campaign, using a Bonferroni adjustment (*P* < 0.025) for multiple ANOVAs. Pearson correlation was used to explore the effect of contemporaneous biotic and abiotic environmental variables, such as physicochemical properties, nutrients, and bacteria and phytoplankton concentrations measured by FCM, on relative abundance of Acantharia clades using rRNA only. Since the goal of the correlation analysis was to evaluate fine-scale effects that could be confounded by the large-scale regional differences already captured by the ANOVA, we performed separate analyses for Canada Basin and Nares Strait, and since many variables were expected to be collinear with depth, we controlled for depth using the residuals of the linear model. All statistical analyses were performed on log-transformed data using the base code of R v. 3.3.3 (R Core Team, 2017).

Weighted Gene Correlation Network Analysis (WGCNA) was performed using the R package of the same name (Langfelder and Horvath, 2008) on rRNA data. The ASV table for WGCNA was trimmed by removing ASVs with mean relative abundance < 10^−5^ and rarefying all samples to 10 664 reads per sample.

## RESULTS

**Fig. 2:**
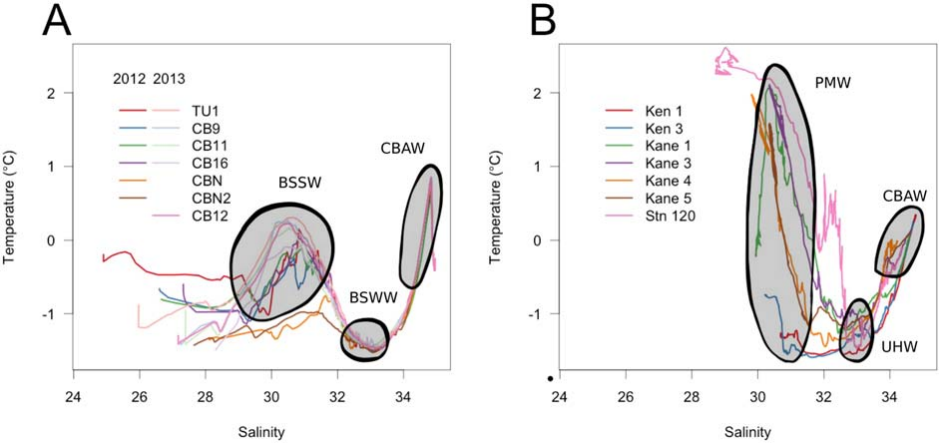
Temperature versus Salinity plots from complete CTD profiles. The water masses identified in the text are indicated by shading. BSSW = Bering Sea Summer Water; BSWW = Bering Sea Winter Water; CBAW = Canada Basin Atlantic Water; PMW = Polar Mode Water; UHW = Upper Halocline Water. (A) Canada Basin and (B) Nares Strait*.*

**Fig. 3:**
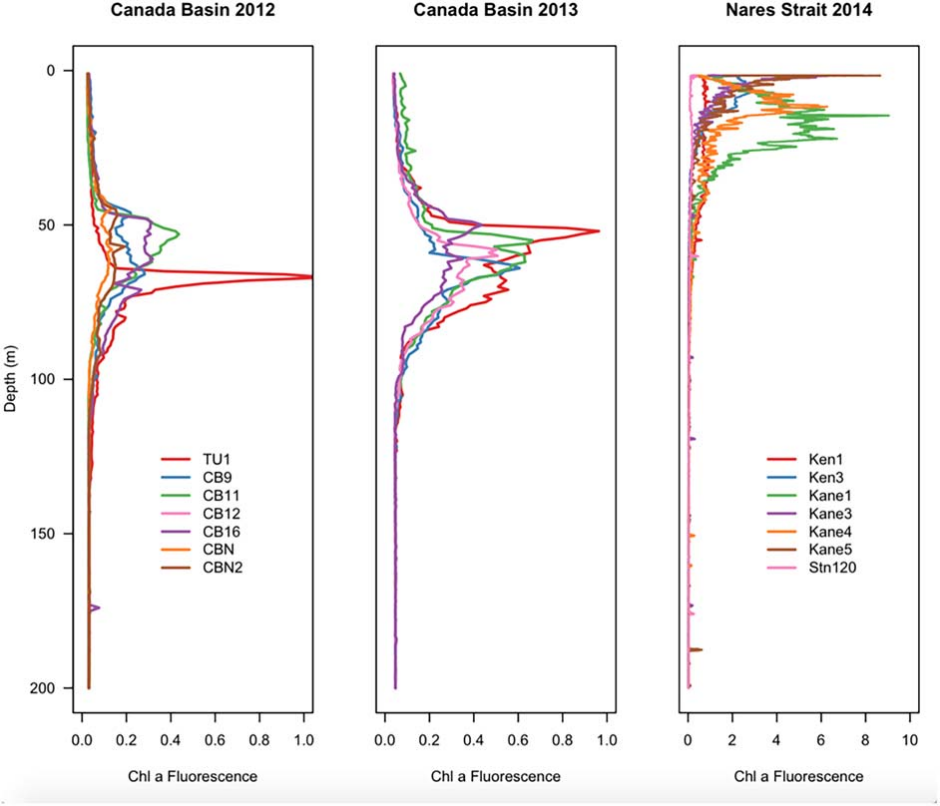
Depth profiles of *in situ* chlorophyll *a* fluorescence measured by CTD-mounted Seapoint Fluorometer in the Canada Basin and Nares Strait in the upper 200 m of the water column. Note different X-axis scale for Nares Strait.

Sampling occurred in late summer (August) in the Canada Basin in 2012 and 2013, and in Nares Strait in 2014 (Fig. 1). The two sampling years in Canada Basin represented contrasting ice conditions. In 2012, owing to a gale event in early August, the majority of stations had experienced ice breakup 9–27 days before sampling (Canadian Ice Service and archived ship log data). Only stations CBN and CBN2 were ice-covered at the time of sampling. In contrast, all stations were ice-covered in 2013 (Fig. 1a). In Nares Strait at the time of sampling, sea ice covered ~ 50% of the water surface in Kennedy Channel, and ≤ 20% of the surface in Kane Basin and at Stn 120 (Fig. 1b). For both regions, water masses were distinguished based on Temperature–Salinity diagrams (Fig. 2), while the SCM was identified based on *in situ* fluorescence (Fig. 3). In the Canada Basin, we sampled the surface mixed layer, the SCM (usually located in the modified Pacific water of the UHW) and the Pacific origin Bering Strait Winter Water (BSWW) (Fig. 2). CBAW occurs below 250–300 m and was not included in our analysis, as DNA concentrations were too low for successful extraction using our standard technique. Physical oceanographic data, nutrients and results of FCM used for correlation analysis are given in Table S2.

ASVs from rRNA samples were used to evaluate the taxonomic composition of the active eukaryote community. The two sampling years in the Canada Basin were broadly similar to each other; dinoflagellate sequences dominated at the surface in all samples, and rhizarians (mostly Acantharia) dominated in Pacific Winter Water (Figs. S1, S2). Chlorophytes reached high relative abundance in surface samples from the < 3 μm size fraction in both years, as well the > 3 μm size fraction in 2013. Also in 2013, diatoms were abundant at the SCM in the large size fraction only (Fig. S2).

Samples from the large fraction in Nares Strait 2014 showed similar high relative abundance of dinoflagellates, while diatoms were prevalent at the surface at all sites. In the < 3 μm size fraction, the relative abundance of higher taxonomic groups in the surface water showed more evenness, with a greater relative abundance of the presumed parasitic Marine Alveolates below 50 m. In northern Baffin Bay (Station 120) dinoflagellates were dominant throughout the water column, except for the deepest (300–550 m) small size fraction in which acantharian Rhizaria and choanoflagellates were relatively more abundant (Fig. S3).

**Fig. 4:**
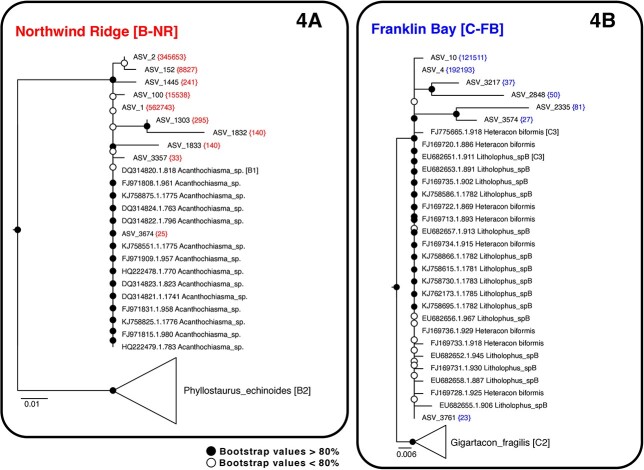
Phylogenetic trees of Acantharian ASVs. Trees were constructed from a comparative analysis of V4 region of 18S rRNA gene (rDNA) from the most common ASVs (total numbers of ASVs in in parenthesis) and available Arctic clone library sequences using the ML method with the K2P substitution model. Rooting clades are based on representative V4 region sequences in Supplementary Fig. S4. Bootstrap support values are expressed as percentages of 1000 replicates. (A) Clade B-NR with 10 ASVs and 40 V4 18S rDNA environmental sequences (233 bp alignment). Scale bars show the number of substitutions per site over the 233 bp alignment. (B) Clade C-FB sequences showing the relationships of eight ASVs and 27 reference sequence. Scale bars show the number of substitutions per site over the length of the 260 bp alignment.

In both regions, acantharians were found at low relative abundance in surface waters, but often dominated eukaryote taxa in the Pacific origin waters, reaching relative abundances of 81% of the total microbial eukaryote rRNA reads in a single sample from the Canada Basin. Acantharia abundance was lower in Nares Strait, reaching maximum relative abundance of 9.2% of total rRNA reads. Notably, Acantharia abundance in Nares Strait was higher in rDNA reads, reaching 35% of total reads (data not shown). Following Decelle *et al.* (Decelle *et al.*, 2013) and the PR2 database (Guillou *et al.*, 2012), the two dominant ASV types were placed into clades B1 and C3. To further explore the taxonomic placement and diversity of the ASVs, we constructed a reference phylogenetic tree using available nearly full length 18S rRNA sequences, including from the Arctic (Fig. S4). The DNA-sourced amplicons were then aligned with the V4 region of sequences in the reference tree and new ASV trees were constructed (Fig. 4). Two clades in the Arctic stood out and we refer to these clades as B-NR and C-FB, in reference to the sampling sources of the Arctic sequences most closely aligned with our ASVs (Fig. 4a and b; Fig. S4).

**Fig. 5:**
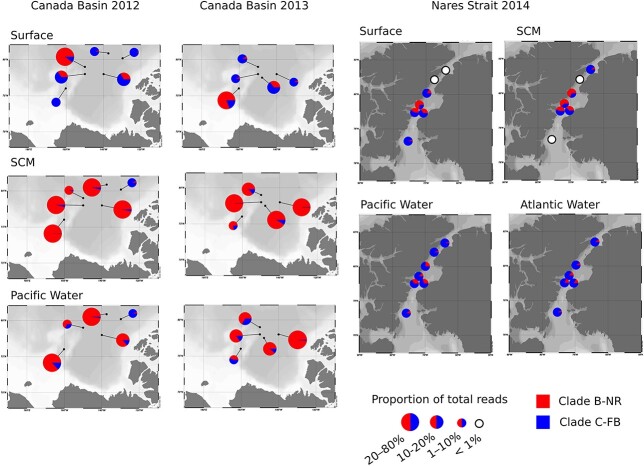
Relative abundance of ASVs in rRNA samples for Acantharia clades B-NR and C-FB. Size fractions have been pooled and multiple sample depths have been pooled for some water masses (maps created using Ocean Data View v. 4.7.4) (Schlitzer, 2015). Station names from Fig. 1.

Of the acantharian reads, 99% belonged to these two clades, with 86% and 13% assigned as B-NR and C-FB, respectively. The two clades’ distribution within water masses differed between the two regions of the Arctic (Fig. 5, showing rRNA only). Clade B-NR was dominant (> 10%) in BSWW (Pacific Water) at most sites in the Canada Basin, but at two sites, CB11 in 2012 and TU1 in 2013, the highest abundance of Clade B-NR occurred in the < 3 μm size fraction at the surface (18.4% and 29.1% of reads, respectively) with lower abundances at the SCM (8.9% and 1.4%) (Fig. 5). For both sites, the other year followed the “typical” pattern of higher abundance at greater depth, and < 10% at the surface. Clade B-NR was also present at very low abundances (< 0.4%) in all depths and size fractions at the site CBN2 in 2012. In contrast, the relative abundance of Clade C-FB was comparable in both Canada Basin and Nares Strait, though it was never above the 10% dominance threshold.

**Fig. 6:**
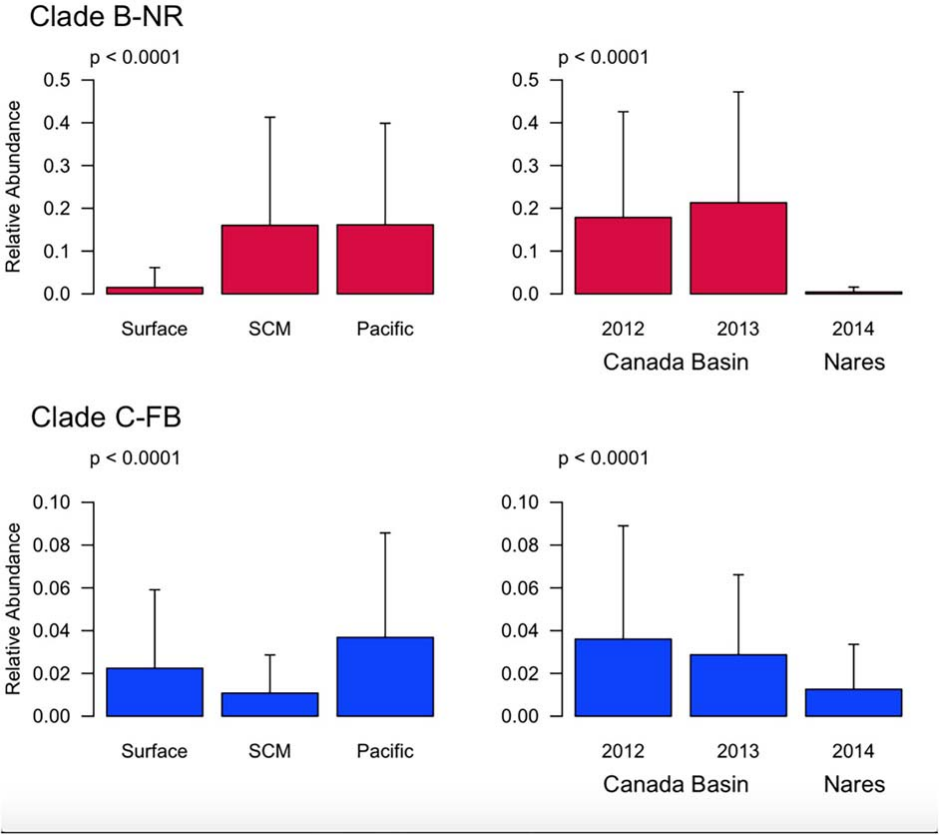
ANOVA on Acantharia clades. Effect of factors Water Mass (left), and Sampling Campaign (right) on relative abundance of the two dominant Acantharia clades. Error bars represent one standard deviation. P values come from ANOVA. Note that because we use a Bonferroni correction for repeated ANOVAs, the threshold of significance is p < 0.025. n.s. = no significance. SCM = Subsurface Chlorophyll Maximum.

ANOVA for both clades showed a significant effect of water mass (Fig. 6; Table S3), with Clade B-NR having lowest abundance in fresher surface water, compared to Clade C-FB with lowest abundance in the Pacific-influenced waters of the SCM. A separate ANOVA of Nares Strait indicated that Clade C-FB was significantly more abundant in CBAW than other water masses (*P* < 0.0001; data not shown). Both clades had lower abundance in Nares Strait than in the Canada Basin, though this effect was more pronounced for Clade B-NR, whose relative abundance in Nares Strait was 0–10% at the surface and SCM, and < 1% in deeper water masses (Fig. 5). Neither clade showed any significant difference between the abundance in 2012 versus 2013 in the Canada Basin (Fig. 6).

The interaction term for Water Mass × Nucleic Acid (rRNA or rDNA) was significant for both Clades B-NR and C-FB (*P* < 0.001). Using paired t tests, we found that Acantharia reads had higher relative abundance in rRNA (*P* = 0.004), except for in the deepest water masses sampled (BSWW in the Canada Basin and CBAW in Nares Strait), where they had higher relative abundance in rDNA (*P* < 0.0001).

Using environmental data (Table S2), we constructed a Pearson correlation matrix with the relative abundances of clades B-NR and C-FB (Table I). Clade B-NR in Nares Strait, where its relative abundance was negligible was not analyzed further. Correlations for Clade C-FB had the same sign in both Canada Basin and Nares Strait, suggesting that we have detected robust ecological trends for this clade, though greater significance levels were seen in the Canada Basin where its relative abundance was higher. While controlling for depth-related variability, relative abundance of Clade B-NR was correlated to markers of a history of greater primary productivity, including high DO and chl *a*, with depleted NO_3_. In contrast, Clade C-FB had a significant positive correlation to NO_3_ and SiO_2_ concentrations, and significant negative correlations to salinity, DO saturation, fluorescent colored dissolved organic material (*f*CDOM), and chl *a* fluorescence, consistent with ecological differences between the two clades. The clades were negatively correlated to each other in the Canada Basin.

**Table I:** Pearson correlation coefficients. Relative abundance of Acantharia clades B-NR and C-FB (in rRNA, size fractions pooled) correlated to contextual environmental data.

Region	Canada Basin	Canada Basin	Nares Strait
Clade	B-NR	C-FB	C-FB
Salinity	0.27	−0.43^*^	−0.21
NO_3_	−0.46^**^	0.48^**^	0.09
PO_4_	0.34	0.30	0.11
SiO_2_	−0.34	0.50^**^	0.01
Dissolved O_2_	0.57^***^	−0.45^*^	−0.24
*f*CDOM	0.22	−0.44^*^	−0.60^*^^*^^*^
Chl a	0.37^*^	−0.56^*^^*^^*^	−0.25
Bacteria	−0.12	0.16	−0.13
Picophyto	−0.12	−0.13	−0.31^*^
Nanophyto	0.26	−0.33	00.01
Clade B-NR		−0.57^*^^*^^*^	
Clade C-FB	−0.57^***^		

Correlations are calculated on residuals of a linear model controlling for sample depth. Clade B-NR correlations are calculated using data from the Canada Basin only, while Clade C-FB correlations are calculated on Canada Basin and Nares Strait data. Pearson Correlation significance indicated by asterisks: ^*^P < 0.05; ^*^^*^P < 0.01; ^*^^*^^*^P < 0.001. Picophytoplankton (Picophyto), Nanophytoplankton (Nanophyto)

To detect the co-occurrence of Acantharia and other taxa, we performed WGCNA, which divided the ASV dataset into 34 modules based on co-occurrence. A total of 642 ASVs, representing 33% of sequences, couldn’t be assigned to a module, including most Clade C-FB sequences (> 99%), suggesting they were ubiquitous. Most Clade B-NR sequences (97%) were found in a module containing a total of 40 ASVs (nine belonging to Clade B-NR). Other ASVs belonging to the same module as Clade B-NR included ciliates, dinoflagellates (including plastid-bearing, heterotrophic, and parasitic taxa), a haptophyte, fungi and polycystine radiolarians (Table S4).

## DISCUSSION

Our results for the whole microbial eukaryote community are consistent with previous studies of the Canada Basin that show species assemblages dominated by ciliates and dinoflagellates (Onda *et al.*, 2017), as well as the higher abundance of diatoms found in Nares Strait and northern Baffin Bay (Kalenitchenko *et al.*, 2019; Freyria *et al.*, 2021). These results highlight the ecological differences between the planktonic communities of these two regions, which have been previously analyzed by Ardyna *et al.* (Ardyna *et al.*, 2011). However, the increased geographic and vertical coverage of our study found high relative abundances (up to 81% of reads) of Acantharia across a broad area of these two Arctic regions, which, to our knowledge, has not been highlighted previously. Our use of molecular techniques also circumvented difficulties associated with the preservation and imaging of specimens that cause Acantharia to be underestimated by traditional methods (Biard *et al.*, 2016). We suggest that another reason this group has been underestimated is because of its association with mesopelagic depths, which have received less attention than the better studied euphotic zone, supported by findings from earlier 18S rRNA clone library studies discussed below.

Phylogenetic analysis showed that 99% of acantharian sequences belonged to two Acantharia clades corresponding to the cyst-forming clades B and C (Decelle *et al.*, 2013). We note that the V9 region, used in the previous phylogeny, was missing from our nearly full-length environmental sequences and is generally less well-represented in environmental surveys compared to the V4 region (Comeau *et al.*, 2011; Tragin *et al.*, 2018). There was some discrepancy in subclade branching order between nearly full sequences and the V9-based trees used in Decelle *et al.* (Decelle *et al.*, 2013) and in the future multigene trees coupled with morphologically identified specimens might be needed to resolve true branching order. The two distinct clades from the Arctic samples were designated as B-NR for the clade aligned to clone library sequence (DQ314821) originally from a SCM sample collected in autumn 2002 near the TU1 station along the Northwind Ridge of the Canada Basin (Lovejoy *et al.*, 2006), and C-FB for the clade aligned to four clones (FJ697XX) from samples collected in 2004 in Franklin Bay in the Southern Beaufort Sea (Terrado *et al.*, 2009). The two clades differed in their geographic distributions. Clade C-FB was found at low but comparable abundances in the Canada Basin and Nares Strait and tended to be more frequent in deeper water masses with Pacific origin signatures. In contrast, Clade B-NR was negligible in Nares Strait, but dominated in the Canada Basin over two sampling years with contrasting ice conditions, suggesting recurring high-abundance events whose duration is not known, but whose reappearance suggests little phenological variability. We assume that it is unlikely our sampling would have missed a late-summer occurrence of Clade B-NR in Nares Strait if it were present, given that sites were sampled in August, when Arctic microbial eukaryotic communities are relatively stable (Comeau *et al.*, 2011; Joli *et al.*, 2017; Freyria *et al.*, 2021).

Acantharia sequences from earlier 18S rRNA clone library studies (Table S5) support our findings that these two clades are recurrent members of Arctic water column communities, being detected in the Canada Basin in five different years going back to 2002 (Lovejoy *et al.*, 2006; Terrado *et al.*, 2009; Lovejoy and Potvin, 2010; Lie *et al.*, 2014; Terrado R, Scarcella K, and Lovejoy, unpublished), as well as in 2005 in Baffin Bay (Hamilton *et al.*, 2008). The development of high-throughput sequencing technology holds the promise of a more complete picture of their distributions, with Clade C-FB repeatedly detected at one site in Baffin Bay from 2008–2016 (Freyria *et al.*, 2021) and Acantharia detected at sites along the continental shelf of the Canada Basin in 2009 (Monier *et al.*, 2013). Overall, however, high-throughput sequencing surveys carried out to date in the Arctic, including the Tara Polar Circle expedition (e.g. Ibarbalz *et al.*, 2023), had limited coverage of Canada Basin and Nares Strait. In addition, Tara results to date are mostly limited to surface and near surface samples with little coverage of the subsurface water column; thus, in light of our results, it is not surprising that the Acantharia would have been easily missed.

We compared the effect of local environmental conditions and water mass history on the distribution of the two Acantharia clades. Since high abundances of clade B-NR were usually (but not always) associated with Pacific sourced waters in the Canada Basin, one parsimonious explanation suggests a Bering Sea water inoculum into the Canada Basin. However, the detection of the same phylotype from the region in autumn 2002 (Lovejoy *et al.*, 2006) and in multiple years after (Fig. 4, Table S5), would be consistent with the clade representing a resident taxon with recurrent late summer–autumn activity determined by local environmental conditions. Correlation with markers of photosynthetic activity supports the notion of seasonal variability with primary productivity. Additionally, WGCNA found that Clade B-NR co-occurred with several other eukaryote taxa (Table S3), a grouping that could suggest either overlapping ecological niches or specific prey or symbiont relationships, as discussed below.

In contrast, the association of Clade C-FB with Pacific-sourced waters, which in the Canada Basin, was supported by a significant positive correlation with nutrients SiO_2_, and NO_3_ (Anderson *et al.*, 2013), were seen in both regions. The occurrence of C-FB in Pacific waters on two sides of the Arctic is consistent with a distribution determined by long-range advective transport. WGCNA found no co-occurring taxa, suggesting passive transport rather than C-FB actively interacting with other microorganisms. This clade was detected year-round at mesopelagic depths in Franklin Bay during an overwintering expedition from 2003–2004 (Fig. S4, sequences FJ169713, FJ169711, FJ169734, FJ169734). This water is part of a Pacific jet that flows along the southern Canadian Beaufort Sea (Williams *et al.*, 2014) and can subsequently be entrained into the Beaufort Gyre (MacKinnon *et al.*, 2021). In contrast to B-NR, the C-FB clade would be more likely to be imported constantly from Pacific waters, and then entrained into the Beaufort Gyre, which sheds water that moves east along the Canadian shelf to eventually arrive in Nares Strait. Modeling estimates of transit time of Pacific Water from Bering Strait to northern Baffin Bay are highly variable but fall within the range of 3–15 years (Hu and Myers, 2013; Aksenov *et al.*, 2015). While further microscopy and experimental data would be needed to test this hypothesis, this heterotrophic clade could be sustained over long dark periods of transport by grazing during windows of prey abundance.

Despite being a recurring feature of Arctic water columns, the distribution of both clades may be impacted by environmental change in the Arctic. Pacific inflow through Bering Strait has increased in recent decades (Woodgate, 2018), which could increase the abundance of taxa such as Clade C-FB associated with this water mass. On the other hand, Clade B-NR may be vulnerable to climate change, as a resident Arctic taxon that appears to respond to seasonal cues. Though we did not detect a change in its distribution between a high-ice and low-ice year, a longer time series may be needed to determine the cumulative effects of increased stratification or invasion of taxa from lower latitudes.

While sequencing data in the present study provide evidence that Acantharia are present in late summer Arctic water columns at ecologically significant abundances, inferences of cellular abundance must be interpreted in light of the complex acantharian life cycle (Suzuki and Not, 2015). Multinucleated vegetative cells, cysts, and single-nucleated swarmers would be expected to differ in copy number of 18S rDNA gene as well as metabolic activity producing 18S rRNA. In principle, the pre-filtration step through a 50 μm mesh should exclude vegetative cells and cysts, which are tens to hundreds of microns in diameter (Decelle *et al.*, 2012b); however, sequences from other multinucleated organisms have also been found overrepresented in environmental DNA (eDNA) (Sandin *et al.*, 2022), possibly from cell breakage. Swarmer cells, which are 2–3 μm diameter (Gilg *et al.*, 2010; Decelle *et al.*, 2012c; Rizos *et al.*, 2024), would also pass through this mesh. Decelle *et al.* (Decelle *et al.*, 2013) have suggested that swarmers are the predominant life stage at mesopelagic and bathypelagic depths. Although the life stage cannot be known with certainty from sequencing data, another clue could be the higher relative abundance in the rDNA compared to rRNA in deeper water masses. While this could be due to free DNA or dead cells, it could also suggest that Acantharia at these depths had lower metabolic activity (resting stages), or lower rRNA: rDNA ratios in the small cells compared to larger cells (Fu and Gong, 2017; Casabianca *et al.*, 2021). As discussed below, knowledge of life stage can inform our interpretation of the vertical distribution of acantharian clades.

Contrasting with the findings of Decelle *et al.* (Decelle *et al.*, 2013), who found that cyst-forming Acantharia clades B and C were distributed evenly through the photic and mesopelagic zone, our analysis showed that both clades were more abundant in deeper water masses. This vertical distribution might result from cyst formation and sinking, which is hypothesized to be a seasonal occurrence in Acantharia, timed to take advantage of phytoplankton bloom detritus (Martin *et al.*, 2010). In the southern Canada Basin, measurements from mooring sediment traps in 2012 and 2013 found that diatom export peaked in July but continued until the end of August and was of comparable magnitude in both years (Dezutter *et al.*, 2019). If the acantharians sink just after the diatoms, then the timing would be congruent with our detection of acantharian swarmer cells in BSWW in late August. As with other rhizarian taxa with biomineralized skeletons (Guidi *et al.*, 2016), cyst-forming acantharians can export carbon, and in the case of Acantharia, sulfate, to the deep ocean, or contribute to benthic–pelagic coupling in shelf and shallow waters. In support of deep export, we note that Lie *et al.* (2014) found B-NR in 500 m water in the Canada Basin (Supplementary Table S5).

Clade B-NR occasionally deviated from this deeper distribution and was found at highest abundance at the surface and not in the deeper samples on two occasions in the Canada Basin (Fig. 5). These surface occurrences did not occur at the same sites over the two years. The magnitude of difference in relative abundances is greater than we would expect from stochastic variability. Notably, such high surface abundances have not previously been observed in the 18S rRNA clone libraries from the Canada Basin, discussed above. Since the acantharian reproductive cycle involves the physical separation of life stages (Martin *et al.*, 2010), we suggest that the different observed distributions could be caused by phenological variability of sinking events, with larger vegetative stages at the surface synchronously forming cysts and sinking to release swarmer cells at deeper depths. Such events are expected in late summer, but their exact timing could vary depending on local conditions. The sites with higher surface abundances of Acantharia were not unique in terms of chl *a* concentration or time since ice break-up, which controls bloom timing in the Arctic (Song *et al.*, 2021), compared to other sites. More sampling is required to capture environmental or stochastic patterns to describe timing of these stages.

There was also some evidence of variability in the vertical distribution of Clade C-FB. By re-examining previously published data from northern Baffin Bay (Freyria *et al.*, 2021), we identified Clade C-FB comprising up to 25% of reads in the SCM in autumn samples, whereas in the late summer data collected in the present study, this clade was much less abundant (< 3% of reads) at the SCM than at other depths (Fig. 6). The sampling site in Freyria *et al.* was located ~ 160 km southeast of our Stn 120 and influenced by the north-flowing West Greenland Current. Further sampling would be needed to determine if variation in depth and abundance of C-FB has a basis in seasonal or geographic and ocean circulation factors.

Following the phylogeny of Decelle *et al.* (Decelle *et al.*, 2013), the genus most closely related to our Clade B-NR is *Acanthochiasma*, which has both traits of being cyst-forming and having the ability to capture and retain taxonomically diverse photosymbionts, including the haptophyte alga *Chrysochromulina* and photosynthetic dinoflagellates (Decelle *et al.*, 2012a). This is unusual among mixotrophic acantharian clades, most of which are non-cyst-forming. The capture and sequestration of photobionts enables a mixotrophic lifestyle and the combination of prey capture and photosynthesis by the symbionts would be advantageous in the low-productivity regime of Canada Basin, which would limit the relative abundance of prey for acantharians, including ciliates, metazoan larvae, diatoms and other acantharians (Swanberg and Caron, 1991). Acantharia have not been mentioned in previous reviews on mixotrophic protists in Arctic waters, e.g. Stoecker and Lavrentyev (Stoecker and Lavrentyev, 2018); however, the general lack of data on these fragile cells would explain this oversight. While imaging would be necessary to determine whether the Arctic Clade B-NR have symbionts or not, the fact that WGCNA found that they co-occurred with potentially photosynthetic dinoflagellate taxa (Table S4) offers a promising avenue of research. If they proved to be mixotrophic, then given their widespread distribution found in this study, we suggest that these relatively large protists could have an impact on food web dynamics and carbon sequestration by packaging surface derived organic carbon into larger acantharian “particles” in the form of cells and cysts that sink.

## CONCLUSIONS

Overall, we found that the null hypothesis that local selection determines the distribution of Acantharia populations in the two regions under study was not supported at least for one of the clades (C-FB). Overall, there was strong evidence that distributions were linked to water masses and circulation patterns. Based on molecular data from the Canada Basin, a potentially mixotrophic clade (B-NR) dominated and accounted for up to 81% of total microbial eukaryotic reads at the depth of the halocline. In light of recurrent records of the same clade in the Canada Basin, occurrences correlated to markers of a history of greater primary productivity, we speculate that the acantharian clade B-NR may be resident and favored by seasonal cycles in the northern part of the Canada Basin. In contrast, Nares Strait between Greenland and Ellesmere Island, was marked by the presence of a most likely strictly heterotrophic clade (C-FB) that has been most often reported in the Arctic in mesopelagic Pacific origin waters along the Beaufort Shelf. In Nares Strait our Clade C-FB Acantharia accounted for 35% of total microbial eukaryote reads in Pacific-influenced UHW of southern Kane Basin and the northernmost part of Baffin Bay. Both clades of acantharians were much rarer nearer the surface in Arctic origin PMW. We suggest that the vertical distributions may be determined by the life cycle stages of these two clades, which produce both sinking cysts and swarmer cells, a characteristic that may also facilitate survival during multi-year transport between the Canada Basin and Nares Strait. While many small planktonic protists are indigenous to the Arctic Ocean, this study illustrates how Pacific origin plankton could be transported across the most northerly regions of the Canadian Arctic to arrive as far south as Pikialasorsuaq (northern Baffin Bay). Such passive advective transport would be a previously undescribed northern route for the transport of microbial species towards the Atlantic. In addition, the ubiquity of Acantharians suggest that they contribute to the food web and strontium-sulfur cycling over much of the Arctic. The effect of current climate trends leading to longer open water seasons and an accelerating hydrological cycle is unknown but could favor the more globally distributed species (Clade C-FB) at the expense of putative Arctic resident species (Clade B-NR).

## Supplementary Material

Supplementary_Table_S1_and_S2_Thaler_fbae051

Supplementary_Materials_Tables_S3_S4_FigS1_4_Thaler__fbae051

Supplementary_Table_S5_Thaler_fbae051

## Data Availability

All sequence reads are deposited under bioprojects PRJNA362945 (Canada Basin) and PRJEB24314 (Nares Strait) in NCBI. Physical and nutrient profiles are available at https://www.whoi.edu/beaufortgyre for the Canada Basin and via Amundsen Science at https://amundsenscience.com/data/data-access/for the Nares Strait and northern Baffin Bay. Fasta files and PR2 taxonomy of all ASVs are available on Figshare: https://doi.org/10.6084/m9.figshare.26370664.v1.
