# Supplementary material for: Environmental selection and advective transport shape the distribution of two cyst-forming Acantharia clades in the Canadian Arctic
Source: J Plankton Res. 2024 Oct 4;46(6):542–54. doi: 10.1093/plankt/fbae051 (PMC11629782; doi:10.1093/plankt/fbae051)
Supplement: Supplementary_Table_S1_and_S2_Thaler_fbae051 [file supplementary_table_s1_and_s2_thaler_fbae051.docx]

**Supplementary Table S1**. Number of raw and filtered (Filt-Merge) 18S rRNA and rDNA reads per sample, not available (na), Large and Small as in text.

| **nucleic acid** | rRNA | rRNA |  | rRNA | rRNA |  |  | rDNA | rDNA |  | rDNA | rDNA |
| --- | --- | --- | --- | --- | --- | --- | --- | --- | --- | --- | --- | --- |
| Size fraction | Large | Large |  | Small | Small |  |  | Large | Large |  | Small | Small |
| **Sample_ID** | Raw | Filt-Merge |  | Raw | Filt-Merge |  | **Sample_ID** | Raw | Filt-Merge |  | Raw | Filt-Merge |
| **Ken1_2m** | 89159 | 30469 |  | 94715 | 31964 |  | **Ken1_2m** | 99362 | 52313 |  | 80301 | 41391 |
| **Ken1_30m** | 89330 | 29162 |  | 104477 | 35790 |  | **Ken1_30m** | 116420 | 58546 |  | 84341 | 42846 |
| **Ken1_47m** | 95639 | 34953 |  | 89355 | 28846 |  | **Ken1_47m** | 115982 | 62612 |  | 75235 | 37916 |
| **Ken1_80m** | 89420 | 26561 |  | 91635 | 27645 |  | **Ken1_80m** | 94654 | 49014 |  | 79189 | 38451 |
| **Ken1_121m** | 88987 | 27579 |  | 58514 | 15843 |  | **Ken1_121m** | 104918 | 52676 |  | 81026 | 40990 |
| **Ken1_253m** | 95665 | 29789 |  | 47808 | 13732 |  | **Ken1_253m** | 123152 | 64590 |  | 62393 | 30400 |
| **Ken1_303m** | 85942 | 28461 |  | 84941 | 23687 |  | **Ken1_303m** | 82044 | 40981 |  | 67120 | 32769 |
| **Ken1_542m** | 94913 | 33379 |  | 90316 | 31571 |  | **Ken1_542m** | 94857 | 49196 |  | 47758 | 23683 |
| **Ken3_1m** | 91098 | 33003 |  | 85126 | 29667 |  | **Ken3_1m** | 90663 | 49167 |  | 83704 | 45483 |
| **Ken3_24m** | 92403 | 32833 |  | 86592 | 28719 |  | **Ken3_24m** | 93828 | 49122 |  | 101659 | 51727 |
| **Ken3_40m** | 93616 | 33454 |  | 76404 | 25576 |  | **Ken3_40m** | 86481 | 44711 |  | 81252 | 39403 |
| **Ken3_101m** | 85310 | 24512 |  | 73691 | 21049 |  | **Ken3_101m** | 86252 | 44052 |  | 83640 | 40805 |
| **Kane1_1m** | 137306 | 45262 |  | 87397 | 30427 |  | **Kane1_1m** | 108548 | 62489 |  | 90513 | 49102 |
| **Kane1_30m** | 88330 | 27993 |  | 93097 | 30710 |  | **Kane1_30m** | 88732 | 48495 |  | 81108 | 40812 |
| **Kane1_50m** | 81095 | 24866 |  | 82254 | 26433 |  | **Kane1_50m** | 93472 | 48691 |  | 76693 | 36570 |
| **Kane1_126m** | 88022 | 27996 |  | 75658 | 23318 |  | **Kane1_126m** | 89316 | 45671 |  | 70612 | 31344 |
| **Kane3_1m** | 101990 | 33348 |  | 79912 | 25294 |  | **Kane3_1m** | 85442 | 43728 |  | na | na |
| **Kane3_12m** | 109152 | 36470 |  | 86618 | 29785 |  | **Kane3_12m** | 93547 | 49178 |  | 86912 | 44617 |
| **Kane3_50m** | 100882 | 33007 |  | 42378 | 13560 |  | **Kane3_50m** | 99342 | 52673 |  | 96546 | 50313 |
| **Kane3_127m** | 100982 | 29831 |  | 72000 | 24583 |  | **Kane3_127m** | 91299 | 49950 |  | 85785 | 44008 |
| **Kane3_162m** | 113895 | 37254 |  | 85006 | 29914 |  | **Kane3_162m** | 87308 | 46710 |  | 87327 | 49497 |
| **Kane4_1m** | 101478 | 31758 |  | 82942 | 25521 |  | **Kane4_1m** | 101478 | 43432 |  | 86031 | 45013 |
| **Kane4_35m** | 94079 | 30791 |  | 101963 | 34629 |  | **Kane4_35m** | 96381 | 50854 |  | 97833 | 52963 |
| **Kane4_50m** | 113970 | 38363 |  | 84777 | 27887 |  | **Kane4_50m** | 80022 | 44921 |  | 90863 | 48180 |
| **Kane4_126m** | 117111 | 33881 |  | 89930 | 27974 |  | **Kane4_126m** | 100557 | 55335 |  | 82415 | 41916 |
| **Kane4_228m** | 118419 | 35515 |  | 84226 | 24049 |  | **Kane4_228m** | 162685 | 92889 |  | 79216 | 41082 |
| **Kane5_1m** | 117965 | 41922 |  | 98918 | 34139 |  | **Kane5_1m** | 96703 | 51560 |  | 67856 | 36354 |
| **Kane5_10m** | 93879 | 32608 |  | 83919 | 28694 |  | **Kane5_10m** | 90546 | 50798 |  | 92689 | 49542 |
| **Kane5_26m** | 110520 | 37108 |  | 45423 | 14820 |  | **Kane5_26m** | 101095 | 58603 |  | 89889 | 49225 |
| **Kane5_51m** | 87623 | 28162 |  | 92713 | 30456 |  | **Kane5_51m** | 88044 | 46713 |  | 94678 | 48268 |
|  |  |  |  |  |  |  |  |  |  |  |  |  |
| **nucleic acid** | RNA | RNA |  | RNA | RNA |  |  | DNA | DNA |  | DNA | DNA |
|  | Large | Large |  | Small | Small |  |  | Large | Large |  | Small | Small |
| **Sample_ID** | Raw | Filt-Merge |  | Raw | Filt-Merge |  | **Sample_ID** | Raw | Filt-Merge |  | Raw | Filt-Merge |
| **Kane5_91m** | 89147 | 29169 |  | 92183 | 30595 |  | **Kane5_91m** | 90685 | 49075 |  | 105888 | 56443 |
| **Kane5_142m** | 92541 | 29203 |  | 88128 | 29242 |  | **Kane5_142m** | 99820 | 58183 |  | 97615 | 50240 |
| **Kane5_162m** | 100298 | 32286 |  | 94756 | 30296 |  | **Kane5_162m** | 94300 | 50915 |  | 89229 | 47307 |
| **Kane5_238m** | 100781 | 33970 |  | 88982 | 29572 |  | **Kane5_238m** | 103305 | 56659 |  | 102586 | 51857 |
| **St120_2m** | 90010 | 25873 |  | 101282 | 41880 |  | **St120_2m** | 94891 | 48006 |  | 92731 | 45445 |
| **St120_20m** | 88187 | 33027 |  | 95376 | 38556 |  | **St120_20m** | 89350 | 49869 |  | 103504 | 54151 |
| **St120_40m** | 98027 | 35499 |  | 97196 | 38713 |  | **St120_40m** | 92443 | 50595 |  | 98682 | 53434 |
| **St120_71m** | 97477 | 33737 |  | 101262 | 41613 |  | **St120_71m** | 86206 | 49491 |  | 95495 | 51037 |
| **St120_101m** | 115368 | 38771 |  | 98471 | 40350 |  | **St120_101m** | 100623 | 59615 |  | 110231 | 57946 |
| **St120_304m** | 110691 | 35369 |  | 37888 | 12968 |  | **St120_304m** | 94854 | 55275 |  | 93027 | 48321 |
| **St120_404m** | 99012 | 30886 |  | 134470 | 43997 |  | **St120_404m** | 94920 | 55332 |  | 96724 | 49723 |
| **St120_549m** | 120785 | 39322 |  | 81315 | 28230 |  | **St120_549m** | 92237 | 55076 |  | 98861 | 55892 |
| **TU1_12_7m** | 135081 | 48966 |  | 171231 | 68295 |  | **TU1_12_7m** | 112280 | 60903 |  | 148860 | 87284 |
| **TU1_12_69m** | 209046 | 97705 |  | 193578 | 95979 |  | **TU1_12_69m** | 170739 | 90969 |  | 144537 | 86546 |
| **TU1_12_93m** | 156836 | 65363 |  | 141223 | 57870 |  | **TU1_12_93m** | 167088 | 117945 |  | 174454 | 129283 |
| **CB9_12_8m** | 147883 | 67142 |  | 110981 | 56957 |  | **CB9_12_8m** | 197248 | 125940 |  | 179345 | 115256 |
| **CB9_12_69m** | 343284 | 176113 |  | 158271 | 78276 |  | **CB9_12_69m** | 75739 | 49465 |  | 145340 | 93516 |
| **CB11_12_7m** | 43613 | 0 |  | 51844 | 34135 |  | **CB11_12_7m** | 22234 | 0 |  | 85465 | 73726 |
| **CB11_12_79m** | 116223 | 78834 |  | 108759 | 81685 |  | **CB11_12_79m** | 105001 | 88520 |  | 85947 | 74674 |
| **CB11_12_105m** | 38445 | 23532 |  | 60241 | 44252 |  | **CB11_12_105m** | 69690 | 59316 |  | 77071 | 66506 |
| **CB16_12_7m** | 146879 | 62533 |  | 115224 | 45377 |  | **CB16_12_7m** | 166279 | 106671 |  | 132395 | 84330 |
| **CB16_12_56m** | 209136 | 98001 |  | 108092 | 44123 |  | **CB16_12_56m** | 127266 | 80944 |  | 172319 | 110867 |
| **CB16_12_117m** | 145867 | 61178 |  | 105744 | 42415 |  | **CB16_12_117m** | 177300 | 114981 |  | 149094 | 96969 |
| **CBN_12_7m** | 228313 | 99567 |  | 186313 | 97229 |  | **CBN_12_7m** | 962212 | 651454 |  | 208410 | 135724 |
| **CBN_12_88m** | 235557 | 115921 |  | 69222 | 35448 |  | **CBN_12_88m** | 179780 | 122318 |  | 197293 | 130405 |
| **CBN_12_153m** | 224434 | 106333 |  | 157840 | 77751 |  | **CBN_12_153m** | 212608 | 139838 |  | 137136 | 89022 |
| **CBN2_12_7m** | 166278 | 60764 |  | 78246 | 29287 |  | **CBN2_12_7m** | 143237 | 87649 |  | 158705 | 99489 |
| **CBN2_12_64m** | 235256 | 96159 |  | 114860 | 43867 |  | **CBN2_12_64m** | 205446 | 132240 |  | 98995 | 61844 |
| **CBN2_12_154m** | 190794 | 77145 |  | 154805 | 56541 |  | **CBN2_12_154m** | 154246 | 93594 |  | 175735 | 103998 |
| **TU1_13_6m** | 91556 | 38676 |  | 145410 | 59832 |  | **TU1_13_6m** | 195227 | 120951 |  | 163570 | 96380 |
| **TU1_13_55m** | 88882 | 35033 |  | 144573 | 58301 |  | **TU1_13_55m** | 256984 | 150368 |  | 40210 | 24493 |
| **TU1_13_116m** | 80708 | 32333 |  | 141271 | 55602 |  | **TU1_13_116m** | 182196 | 119001 |  | 82693 | 55658 |
| **CB9_13_6m** | 116036 | 50616 |  | 157542 | 57841 |  | **CB9_13_6m** | 179534 | 107383 |  | 559938 | 373349 |
| **nucleic acid** | RNA | RNA |  | RNA | RNA |  |  | DNA | DNA |  | DNA | DNA |
|  | Large | Large |  | Small | Small |  |  | Large | Large |  | Small | Small |
| **Sample_ID** | Raw | Filt-Merge |  | Raw | Filt-Merge |  | **Sample_ID** | Raw | Filt-Merge |  | Raw | Filt-Merge |
| **CB9_13_66m** | 148288 | 74379 |  | 186049 | 81106 |  | **CB9_13_66m** | 164467 | 102737 |  | 183964 | 122596 |
| **CB9_13_117m** | 95564 | 42380 |  | 175440 | 71438 |  | **CB9_13_117m** | 202928 | 132533 |  | 92532 | 66191 |
| **CB11_13_5m** | 98411 | 58326 |  | 84224 | 53676 |  | **CB11_13_5m** | 49647 | 38304 |  | 71574 | 57475 |
| **CB11_13_84m** | 52923 | 31150 |  | 121590 | 78460 |  | **CB11_13_84m** | 52455 | 41458 |  | 75511 | 60914 |
| **CB11_13_114m** | 94970 | 59785 |  | 58887 | 36943 |  | **CB11_13_114m** | 47271 | 40438 |  | 79952 | 66868 |
| **CB12_13_6m** | 118815 | 52737 |  | 119370 | 49365 |  | **CB12_13_6m** | 173007 | 110928 |  | 212285 | 136084 |
| **CB12_13_63m** | 112662 | 52304 |  | 157230 | 65157 |  | **CB12_13_63m** | 191376 | 122533 |  | 216726 | 131692 |
| **CB12_13_116m** | 134695 | 69271 |  | 176992 | 74705 |  | **CB12_13_116m** | 195833 | 125334 |  | 194588 | 123610 |
| **CB16_13_6m** | 125573 | 56461 |  | 86840 | 37195 |  | **CB16_13_6m** | 187246 | 122927 |  | 188025 | 124280 |
| **CB16_13_53m** | 113299 | 48620 |  | 189578 | 89435 |  | **CB16_13_53m** | 142676 | 83408 |  | 163471 | 95928 |
| **CB16_13_108m** | 131425 | 62412 |  | 142558 | 63932 |  | **CB16_13_108m** | 180321 | 119058 |  | 94774 | 58917 |
|  |  |  |  |  |  |  |  |  |  |  |  |  |

**Supplementary Table S2**.

Summary of environmental data used in this study. Sample ID (Stn-depth) are from Fig. 1 main text: depth is in meters (m), salinity is unitless (Sal), nitrate, phosphate and silicate in μM, oxygen as percent saturation at depth, *fCDOM* in relative fluorescence units (see methods), chlorophyll a concentrations in mg m^–3^, estimated from relative fluorescence. Cell counts from flow cytometry for bacteria (Bact) in 10^5^ cells mL^–1^, both picophytoplankton (PPP) and nanophytoplankton (NPP) in cells mL^–1.^. Not available (na), below detection (nd)

| Sample_ID | Latitude | Longitude | Date | Temp | Sal | NO3 | PO4 | SiO2 | O2 | fCDOM | Chla | Bact | PPP | NPP |
| --- | --- | --- | --- | --- | --- | --- | --- | --- | --- | --- | --- | --- | --- | --- |
| Stn-depth | °North | °East | yy-mm-dd | ° C |  | µM | µM | µM | % sat |  | mg m^–3^ | 10^5^ mL^–1^ | cells mL^–1^ | cells mL^–1^ |
| Ken1_2m | 81.3669 | -63.9571 | 14-08-03 | -1.29 | 30.73 | 1.40 | 0.51 | 5.56 | 89 | 11.10 | 0.42 | 1.66 | 720 | 220 |
| Ken1_30m | 81.3669 | -63.9571 | 14-08-03 | -1.29 | 30.95 | 1.98 | 0.58 | 7.06 | 89 | 10.91 | 0.55 | 1.55 | 430 | 160 |
| Ken1_47m | 81.3669 | -63.9571 | 14-08-03 | -1.23 | 31.19 | 3.05 | 0.70 | 9.10 | 87 | 10.46 | 0.59 | 1.52 | 3830 | 610 |
| Ken1_80m | 81.3669 | -63.9571 | 14-08-03 | -1.51 | 32.52 | 7.66 | 1.09 | 17.07 | 74 | 9.41 | 0.12 | 0.84 | 120 | 30 |
| Ken1_121m | 81.3669 | -63.9571 | 14-08-03 | -1.00 | 33.98 | 10.29 | 0.93 | 11.23 | 69 | 7.22 | 0.02 | 0.69 | 310 | 30 |
| Ken1_253m | 81.3669 | -63.9571 | 14-08-03 | 0.16 | 34.68 | 11.77 | 0.90 | 8.13 | 67 | 4.58 | 0.01 | 0.67 | 670 | 290 |
| Ken1_303m | 81.3669 | -63.9571 | 14-08-03 | 0.23 | 34.71 | 11.84 | 0.89 | 8.03 | 67 | 4.57 | 0.01 | 0.47 | 310 | 140 |
| Ken1_542m | 81.3669 | -63.9571 | 14-08-03 | 0.33 | 34.78 | 12.05 | 0.91 | 7.87 | 69 | 4.40 | 0.01 | 0.45 | 460 | 210 |
| Ken3_1m | 80.7955 | 67.3011 | 14-08-04 | -0.75 | 30.26 | 0.23 | 0.38 | 3.34 | 94 | 10.09 | 1.24 | 1.87 | 190 | 60 |
| Ken3_24m | 80.7955 | 67.3011 | 14-08-04 | -0.92 | 30.66 | 0.87 | 0.48 | 5.20 | 93 | 10.34 | 1.96 | 1.51 | 5820 | 1170 |
| Ken3_40m | 80.7955 | 67.3011 | 14-08-04 | -1.49 | 31.13 | 2.47 | 0.63 | 7.93 | 89 | 11.25 | 0.29 | 1.15 | 1520 | 2000 |
| Ken3_101m | 80.7955 | 67.3011 | 14-08-04 | -1.20 | 33.63 | 10.37 | 1.10 | 17.11 | 69 | 7.47 | 0.02 | 0.62 | 350 | 90 |
| Kane1_1m | 79.9980 | -69.7569 | 14-08-04 | 0.05 | 29.96 | 0.19 | 0.34 | na | 94 | 9.35 | 0.38 | 2.17 | 90 | 20 |
| Kane1_30m | 79.9980 | -69.7569 | 14-08-04 | -0.61 | 31.93 | 1.01 | 0.46 | 2.78 | 94 | 9.25 | 4.26 | 1.59 | 2640 | 3730 |
| Kane1_50m | 79.9980 | -69.7569 | 14-08-04 | -1.22 | 32.67 | 8.01 | 0.94 | 12.92 | 77 | 8.55 | 0.81 | 1.02 | 180 | 30 |
| Kane1_126m | 79.9980 | -69.7569 | 14-08-04 | -0.45 | 34.28 | 10.98 | 0.91 | 10.13 | 69 | 5.66 | 0.02 | 0.69 | 1540 | 2120 |
| Kane3_1m | 79.3501 | -71.8651 | 14-08-05 | 1.90 | 30.45 | 0.17 | 0.28 | 0.99 | 93 | 8.25 | 0.90 | 2.07 | 240 | 70 |
| Kane3_12m | 79.3501 | -71.8651 | 14-08-05 | 1.11 | 31.17 | 0.39 | 0.34 | 1.51 | 100 | 8.66 | 1.07 | 2.17 | 340 | 40 |
| Kane3_50m | 79.3501 | -71.8651 | 14-08-05 | -1.23 | 33.13 | 9.52 | 1.10 | 15.99 | 73 | 7.83 | 0.13 | 1.37 | 750 | 1450 |
| Kane3_127m | 79.3501 | -71.8651 | 14-08-05 | -0.26 | 33.98 | 11.57 | 1.02 | 13.13 | 67 | 5.53 | 0.02 | 1.40 | 110 | 30 |
| Kane3_162m | 79.3501 | -71.8651 | 14-08-05 | 0.12 | 34.63 | 11.97 | 0.91 | 8.99 | 69 | 4.99 | 0.02 | 0.94 | 650 | 300 |
| Kane4_1m | 79.0062 | -70.4914 | 14-08-05 | 1.27 | 29.98 | 0.17 | 0.29 | 2.25 | 94 | 8.60 | 0.22 | 2.05 | 1210 | 410 |
| Kane4_35m | 79.0062 | -70.4914 | 14-08-05 | -1.13 | 31.52 | 2.60 | 0.63 | 8.48 | 90 | 10.4 | 3.23 | 1.08 | 1280 | 700 |
| Kane4_50m | 79.0062 | -70.4914 | 14-08-05 | -1.44 | 32.05 | 5.00 | 0.76 | 9.83 | 89 | 10.0 | 0.6 | 1.88 | 160 | 30 |
| Kane4_126m | 79.0062 | -70.4914 | 14-08-05 | -0.79 | 33.61 | 10.85 | 1.03 | 13.97 | 70 | 6.07 | 0.03 | 2.05 | 220 | 40 |
| Kane4_228m | 79.0062 | -70.4914 | 14-08-05 | -0.03 | 34.00 | 12.38 | 1.09 | 15.47 | 66 | 5.35 | 0.03 | 1.66 | 1460 | 2650 |
|  |  |  |  |  |  |  |  |  |  |  |  |  |  |  |
| Sample_ID | Latitude | Longitude | Date | Temp | Sal | NO3 | PO4 | SiO2 | O2 | fCDOM | Chla | Bact | PPP | NPP |
| Stn-depth  (m) | °North | °East |  | ° C |  | µM | µM | µM | % sat |  | mg m^–3^ | 10^5^ mL^–1^ | cells mL^–1^ | cells mL^–1^ |
| Kane5_1m | 79.0009 | -73.2022 | 14-08-06 | 1.44 | 30.29 | 0.26 | 0.31 | 0.96 | 99 | 8.67 | 1.21 | 2.02 | 130 | 10 |
| Kane5_10m | 79.0009 | -73.2022 | 14-08-06 | 0.82 | 30.69 | 0.40 | 0.37 | 2.07 | 100 | 9.04 | 1.42 | 0.94 | 1090 | 410 |
| Kane5_26m | 79.0009 | -73.2022 | 14-08-06 | -1.13 | 31.75 | 3.06 | 0.65 | 7.57 | 90 | 10.33 | 5.59 | 1.05 | 270 | 100 |
| Kane5_51m | 79.0009 | -73.2022 | 14-08-06 | -1.17 | 32.71 | 7.75 | 0.94 | 12.95 | 79 | 8.61 | 0.21 | 1.12 | 180 | 40 |
| Kane5_91m | 79.0009 | -73.2022 | 14-08-06 | -0.9 | 33.56 | 9.55 | 1.02 | 13.92 | 73 | 6.78 | 0.04 | 1.44 | 300 | 70 |
| Kane5_142m | 79.0009 | -73.2022 | 14-08-06 | -0.55 | 33.84 | 11.15 | na | na | 70 | 5.98 | 0.02 | 1.46 | 220 | 160 |
| Kane5_162m | 79.0009 | -73.2022 | 14-08-06 | -0.28 | 33.95 | 11.52 | 1.04 | 13.66 | 69 | 5.78 | 0.02 | 1.60 | 1950 | 620 |
| Kane5_238m | 79.0009 | -73.2022 | 14-08-06 | 0.09 | 34.60 | 11.54 | 0.91 | 9.20 | 70 | 4.89 | 0.02 | 0.97 | 350 | 80 |
| St120_2m | 77.3228 | -75.7026 | 14-08-06 | 2.27 | 28.89 | 0.25 | 0.31 | 0.76 | 90 | 6.46 | 0.19 | 5.54 | 1750 | 640 |
| St120_20m | 77.3228 | -75.7026 | 14-08-06 | 0.28 | 31.76 | 0.58 | 0.53 | 2.58 | 93 | 9.03 | 0.07 | 2.95 | 1150 | 350 |
| St120_40m | 77.3228 | -75.7026 | 14-08-06 | 0.29 | 32.29 | 1.63 | 0.57 | 4.61 | 89 | 7.66 | 0.13 | 5.00 | 290 | 40 |
| St120_71m | 77.3228 | -75.7026 | 14-08-06 | -1.36 | 32.71 | 5.82 | 0.93 | 11.04 | 83 | 7.79 | 0.05 | 1.67 | 130 | 80 |
| St120_101m | 77.3228 | -75.7026 | 14-08-06 | -1.43 | 33.10 | 7.89 | 0.99 | 12.94 | 81 | 7.02 | 0.03 | 1.80 | 40 | 20 |
| St120_304m | 77.3228 | -75.7026 | 14-08-06 | -0.37 | 34.17 | 11.06 | 0.98 | 12.02 | 71 | 5.69 | 0.02 | 1.09 | 4110 | 1310 |
| St120_404m | 77.3228 | -75.7026 | 14-08-06 | -0.23 | 34.29 | 11.28 | 0.96 | 11.16 | 71 | 5.55 | 0.02 | 1.08 | 2380 | 730 |
| St120_549m | 77.3228 | -75.7026 | 14-08-06 | -0.11 | 34.38 | 11.47 | 0.96 | 11.24 | 71 | 5.34 | 0.01 | 1.19 | 920 | 290 |
| TU1_12_7m | 76.0162 | -160.2448 | 12-08-30 | -0.29 | 25.26 | nd | 0.50 | 2.50 | 78 | 3.07 | 0.04 | 2.68 | 1660 | 193 |
| TU1_12_69m | 76.0162 | -160.2448 | 12-08-30 | 0.04 | 31.20 | 1.84 | 0.93 | 6.29 | 80 | 4.60 | 0.45 | 1.27 | 1092 | 521 |
| TU1_12_93m | 76.0162 | -160.2448 | 12-08-30 | -0.73 | 31.86 | 6.55 | 1.32 | 13.81 | 72 | 5.15 | 0.15 | 1.53 | 203 | 138 |
| CB9_12_8m | 78.0047 | -149.9817 | 12-08-29 | -0.67 | 26.62 | 0.01 | 0.57 | 2.65 | 84 | 3.16 | 0.05 | 2.12 | 1136 | 67 |
| CB9_12_69m | 78.0047 | -149.9817 | 12-08-29 | -0.55 | 31.66 | 4.85 | 1.15 | 10.47 | 76 | 4.77 | 0.3 | 1.47 | 921 | 345 |
| CB11_12_7m | 79.0098 | -149.9880 | 12-08-25 | -0.8 | 26.66 | nd | 0.55 | 2.60 | 83 | 3.20 | 0.03 | 2.01 | 1367 | 109 |
| CB11_12_79m | 79.0098 | -149.9880 | 12-08-25 | -0.71 | 31.89 | 6.70 | 1.30 | 13.13 | 73 | 4.87 | 0.16 | 0.78 | 304 | 122 |
| CB11_12_105m | 79.0098 | -149.9880 | 12-08-25 | -1.3 | 32.32 | 11.10 | 1.63 | 23.18 | 65 | 5.19 | 0.04 | 0.98 | 44 | 29 |
| CB16_12_7m | 77.9965 | -140.0070 | 12-08-29 | -0.71 | 27.35 | nd | 0.57 | 2.36 | 83 | 3.38 | 0.07 | 2.21 | 1217 | 120 |
| CB16_12_56m | 77.9965 | -140.0070 | 12-08-29 | -0.26 | 31.3 | 2.08 | 0.96 | 6.60 | 80 | 4.71 | 0.62 | 1.40 | 1962 | 870 |
| CB16_12_117m | 77.9965 | -140.0070 | 12-08-29 | -1.27 | 32.36 | 11.42 | 1.66 | 24.09 | 66 | 5.18 | 0.05 | 0.92 | 34 | 16 |
| CBN_12_7m | 80.8813 | -137.4445 | 12-08-26 | -1.35 | 28.32 | nd | 0.64 | 2.74 | 85 | 3.20 | 0.05 | 3.45 | 1401 | 104 |
| CBN_12_88m | 80.8813 | -137.4445 | 12-08-26 | -1.35 | 32.35 | 12.19 | 1.74 | 26.34 | 68 | 5.21 | 0.08 | 1.32 | 65 | 18 |
| CBN_12_153m | 80.8813 | -137.4445 | 12-08-26 | -1.44 | 33.15 | 16.19 | 2.01 | 39.5 | 58 | 5.16 | 0.05 | 1.23 | na | na |
| CBN2_12_7m | 80.1997 | -129.8483 | 12-08-28 | -1.43 | 28.78 | 0.19 | 0.66 | 2.83 | 84 | 3.11 | 0.06 | 1.61 | 944 | 101 |
|  |  |  |  |  |  |  |  |  |  |  |  |  |  |  |
| Sample_ID | Latitude | Longitude | Date | Temp | Sal | NO3 | PO4 | SiO2 | O2 | fCDOM | Chla | Bact | PPP | NPP |
| Stn-depth | ° North | ° East |  | ° C |  | µM | µM | µM | % sat |  | mg m^–3^ | 10^5^ mL^–1^ | cells mL^–1^ | cells mL^–1^ |
| CBN2_12_64m | 80.1997 | -129.8483 | 12-08-28 | -1.17 | 31.95 | 7.52 | 1.35 | 14.83 | 69 | 4.89 | 0.28 | 1.54 | 745 | 272 |
| CBN2_12_154m | 80.1997 | -129.8483 | 12-08-28 | -1.52 | 33.18 | 16.02 | 1.97 | 37.35 | 58 | 5.25 | 0.05 | 0.81 | na | na |
| TU1_13_6m | 75.9908 | -160.1075 | 13-08-17 | -1.18 | 26.28 | 0.07 | 0.54 | 2.79 | 83 | 2.89 | 0.04 | 0.42 | nd | nd |
| TU1_13_55m | 75.9908 | -160.1075 | 13-08-17 | 0.17 | 31.16 | 1.68 | 0.91 | 6.50 | 80 | 4.22 | 0.96 | 0.28 | 420 | 250 |
| TU1_13_116m | 75.9908 | -160.1075 | 13-08-17 | -1.18 | 32.35 | 11.19 | 1.62 | 23.03 | 66 | 4.87 | 0.04 | 0.43 | 130 | 110 |
| CB9_13_6m | 78.0232 | -150.1078 | 13-08-21 | -1.38 | 27.27 | 0.01 | 0.58 | 2.56 | 84 | 2.92 | 0.05 | 0.63 | 50 | 10 |
| CB9_13_66m | 78.0232 | -150.1078 | 13-08-21 | 0.05 | 31.08 | 1.17 | 0.87 | 5.57 | 82 | 4.17 | 0.43 | 0.36 | 290 | 290 |
| CB9_13_117m | 78.0232 | -150.1078 | 13-08-21 | -1.22 | 32.29 | 10.76 | 1.60 | 22.18 | 66 | 4.81 | 0.05 | 0.39 | 210 | 50 |
| CB11_13_5m | 78.8677 | -149.9550 | 13-08-21 | -1.29 | 27.78 | 0.04 | 0.59 | 2.52 | 85 | 3.01 | 0.06 | 1.05 | 210 | 10 |
| CB11_13_84m | 78.8677 | -149.9550 | 13-08-21 | -0.83 | 31.92 | 7.88 | 1.36 | 14.8 | 70 | 4.56 | 0.13 | 0.91 | 800 | 600 |
| CB11_13_114m | 78.8677 | -149.9550 | 13-08-21 | -1.27 | 32.33 | 11.55 | 1.64 | 24.15 | 66 | 4.80 | 0.04 | 0.72 | 100 | 80 |
| CB12_13_6m | 77.5160 | -147.8198 | 13-08-22 | -1.40 | 27.20 | -0.04 | 0.56 | 2.46 | 84 | 2.93 | 0.05 | 0.43 | 40 | nd |
| CB12_13_63m | 77.5160 | -147.8198 | 13-08-22 | -0.07 | 31.13 | 1.34 | 0.89 | 5.59 | 81 | 4.27 | 0.61 | 0.32 | 970 | 450 |
| CB12_13_116m | 77.5160 | -147.8198 | 13-08-22 | -1.16 | 32.35 | 11.2 | 1.62 | 23.08 | 66 | 4.93 | 0.04 | 0.36 | 450 | 250 |
| CB16_13_6m | 77.9265 | -140.1597 | 13-08-23 | -1.49 | 28.32 | 0.02 | 0.60 | 2.78 | 85 | 3.06 | 0.05 | 1.11 | 130 | 70 |
| CB16_13_53m | 77.9265 | -140.1597 | 13-08-23 | -0.33 | 31.18 | 1.66 | 0.90 | 6.46 | 81 | 4.06 | 0.42 | 0.57 | 2140 | 780 |
| CB16_13_108m | 77.9265 | -140.1597 | 13-08-23 | -1.24 | 32.33 | 11.47 | 1.63 | 24.08 | 67 | 4.81 | 0.06 | 0.53 | 160 | 40 |
