# Supplementary material for: Environmental selection and advective transport shape the distribution of two cyst-forming Acantharia clades in the Canadian Arctic
Source: J Plankton Res. 2024 Oct 4;46(6):542–54. doi: 10.1093/plankt/fbae051 (PMC11629782; doi:10.1093/plankt/fbae051)
Supplement: Supplementary_Materials_Tables_S3_S4_FigS1_4_Thaler__fbae051 [file supplementary_materials_tables_s3_s4_figs1_4_thaler__fbae051.doc]

**Supplementary materials for Journal of Plankton Research:**

**Environmental selection and advective transport shape the distribution of two cyst-forming Acantharia clades in the Canadian Arctic.**

Mary Thaler, Aurélie Labarre, Connie Lovejoy

Supplementary Tables S1, S2 and S5 are provided separately

**In this document please find:**

**Supplementary Table S3**. Analyses of variance (ANOVAs) using relative abundance of acantharian clades B-NR and C-FB as dependent variables and Nucleic Acid, Size Fraction, Water Mass, and Sampling Campaign as independent variables. Separate analyses were performed for dependent variables, with a Bonferroni correction for multiple comparisons (p < 0.025).

**Supplementary Table S4.** Taxonomy of Amplicon Sequence Variants (ASVs) which co-occurred with Acantharia Clade B-NR, determined by Weighted Gene Correlation Network Analysis (WGCNA).

**Supplementary Figure S1.** Relative abundances of major taxa detected in Canada Basin in 2012 from 18S rDNA amplicon sequencing.

**Supplementary Figure S2.** Relative abundances of major taxa detected in Canada Basin in 2013 from 18S rDNA amplicon sequencing.

**Supplementary Figure S3.** Relative abundances of major taxa detected in Nares Strait in 2014 from 18S rDNA amplicon sequencing.

**Supplementary Figure S4.** Maximum likelihood (ML) phylogenetic tree of Acantharia inferred from 18S rRNA gene sequences from the PR2 database (Guillou *et al.* 2012), and including nearly full length 18S rDNA sequences from environmental NCBI entries. The tree was constructed using the TIM2+F+R4 substitution model in IQTREE. Percentage support from 1,000 pseudoreplicates are shown at the nodes with diamonds. Tips are labelled with NCBI GenBank accession number, taxon name, and clade in square brackets following Decelle *et al.* (2012b). Scale bar shows number of substitutions per site over a total sequence length of 1058 base pairs. Sequences from the Arctic are highlighted with grey boxes. See Supplementary Table S5 for details.

**Supplementary Table S3.** Analyses of variance using relative abundance of acantharian clades B-NR and C-FB as dependent variables and Nucleic Acid, Size Fraction, Water Mass, and Sample Campaign as independent variables. Separate analyses were performed for dependent variables, with a Bonferroni correction for multiple comparisons (p < 0.025).

|  | **Degrees of Freedom** | **Sum of Squares** | **Mean Squares** | **F value** | **p value** |
| --- | --- | --- | --- | --- | --- |
| **Clade B1** |  |  |  |  |  |
| Nucleic Acid | 1 | 0.003 | 0.0028 | 0.154 | 0.69 |
| Size Fraction | 1 | 0.033 | 0.0331 | 1.793 | 0.18 |
| Water Mass | 3 | 1.699 | 0.5665 | 30.672 | < 0.0001 |
| Campaign | 2 | 1.972 | 0.9858 | 53.376 | < 0.0001 |
| Nucleic Acid × Size Fraction | 1 | 0.002 | 0.0024 | 0.131 | 0.72 |
| Nucleic Acid × Water Mass | 3 | 0.436 | 0.1454 | 7.870 | < 0.0001 |
| Size Fraction × Water Mass | 3 | 0.083 | 0.0278 | 1.507 | 0.21 |
| Nucleic Acid × Campaign | 2 | 0.010 | 0.0051 | 0.274 | 0.76 |
| Size Fraction × Campaign | 2 | 0.074 | 0.0371 | 2.010 | 0.14 |
| Water Mass × Campaign | 4 | 1.062 | 0.2655 | 14.374 | < 0.0001 |
| **Clade C3** |  |  |  |  |  |
| Nucleic Acid | 1 | 0.0150 | 0.01503 | 6.896 | 0.009 |
| Size Fraction | 1 | 0.0142 | 0.01415 | 6.492 | 0.011 |
| Water Mass | 3 | 0.1294 | 0.04313 | 19.783 | < 0.0001 |
| Campaign | 2 | 0.0277 | 0.01386 | 6.355 | 0.002 |
| Nucleic Acid × Size Fraction | 1 | 0.0250 | 0.02495 | 11.446 | 0.001 |
| Nucleic Acid × Water Mass | 3 | 0.1851 | 0.06171 | 28.307 | < 0.0001 |
| Size Fraction × Water Mass | 3 | 0.0499 | 0.01664 | 7.631 | < 0.0001 |
| Nucleic Acid × Campaign | 2 | 0.0004 | 0.00020 | 0.093 | 0.91 |
| Size Fraction × Campaign | 2 | 0.0011 | 0.00055 | 0.251 | 0.78 |
| Water Mass × Campaign | 4 | 0.0073 | 0.00183 | 0.841 | 0.50 |

**Supplementary Table S4. Taxonomy of Amplicon Sequence Variants which co-occurred with Acantharia Clade B-NR, determined by Weighted Gene Correlation Network Analysis (WGCNA). Taxa with potential photosynthetic ability are marked with a “*”**

| **Major Group** | **Family** | **Species or Clade** |
| --- | --- | --- |
| Ciliate | Discoctrichidae | NASSO1 |
|  | Strombidiidae | Strombidiidae G |
| Dinoflagellate | Gymnodiniaceae | *Amphidinium longum* |
|  |  | *Ankistrodinium semilunatum* |
|  |  | *Gymnodinium* sp. * |
|  |  | *Gyrodinium dominans* |
|  | Kareniaceae | *Karenia selliformis** |
|  | Warnowiaceae | *Warnowia* sp. |
|  | Amphidomataceae | *Amphidoma languida** |
|  | Thoracosphaeraceae | *Pentapharsodinium tyrrhenicum** |
|  |  | *Scripsiella* sp.* |
|  | Prorocentraceae | *Prorocentrum* sp.* |
|  |  | *Prorocentrum donghaiense** |
|  | Syndiniales | Group I Clade 5 |
|  |  | Group II Clade 3 |
|  |  | Group II Clade 9 |
|  |  | Group II Clade 22 |
|  |  | Group III |
| Haptophyte | Pavlomulinaceae | *Pavlomulina ranunculiformis** |
| Fungi | Sordariomycetes | *Paecilomyces variotii* |
|  | Cystobasidiomycetes | *Rhodotorula mucilaginosa* |
| Radiolaria | Cannobotryidae |  |

**
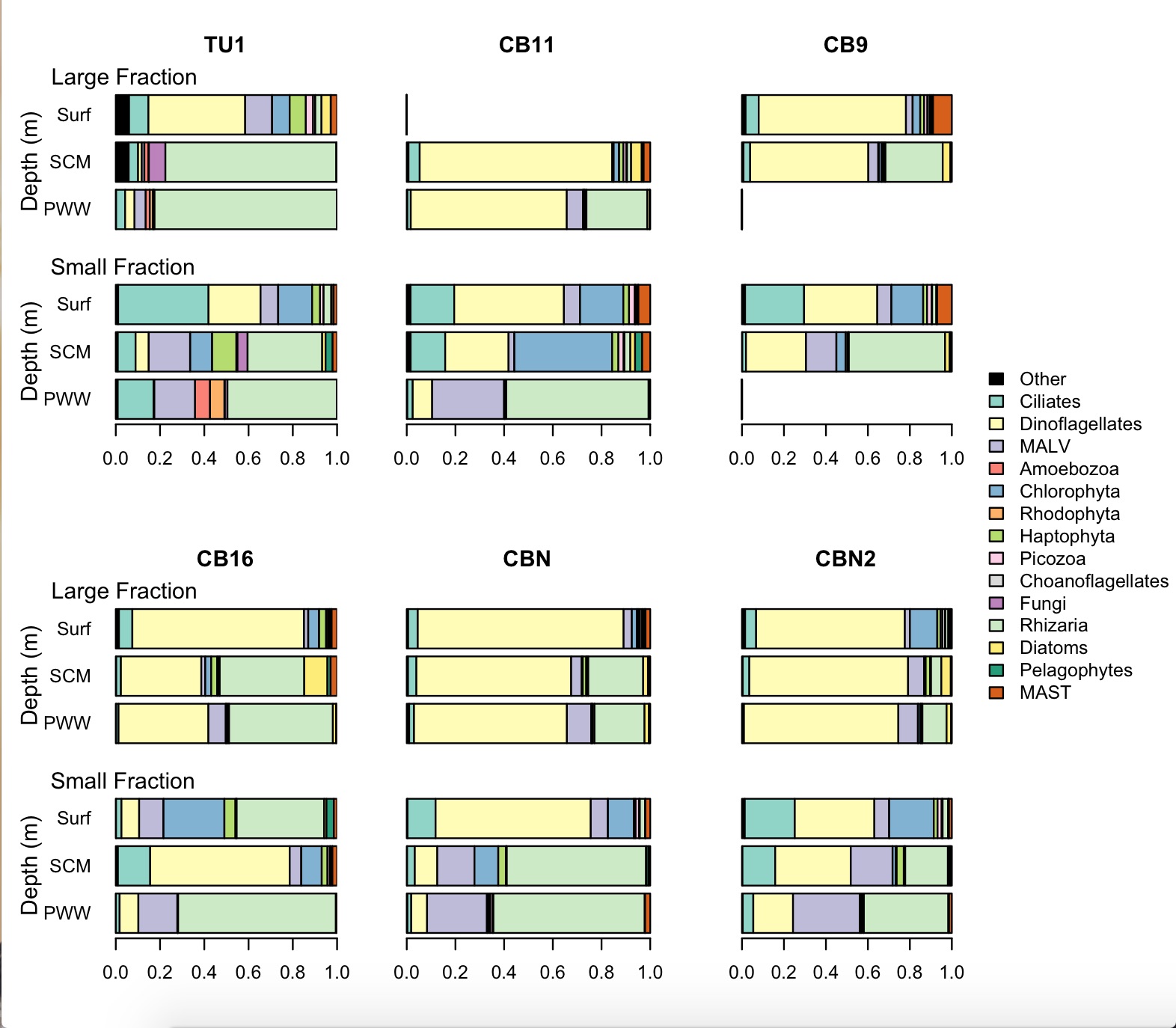
**

**Supplementary Figure S1.** Relative abundances of microbial eukaryotic taxa detected in Canada Basin in 2012 from 18S rDNA amplicon sequencing.

**
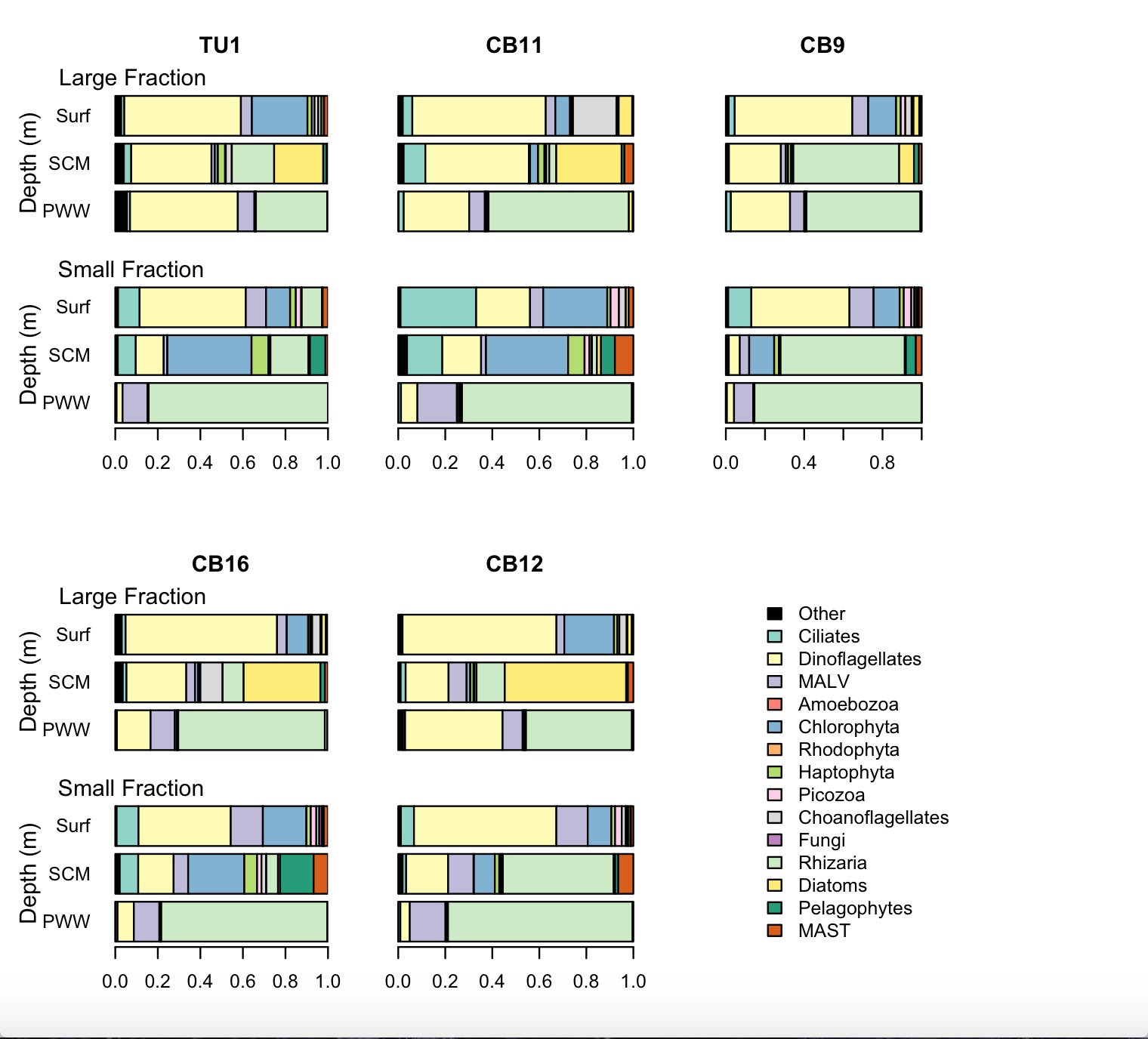
**

**Supplementary Figure S2.** Relative abundances of microbial eukaryotic taxa detected in Canada Basin in 2013 from 18S rDNA amplicon sequencing.

**
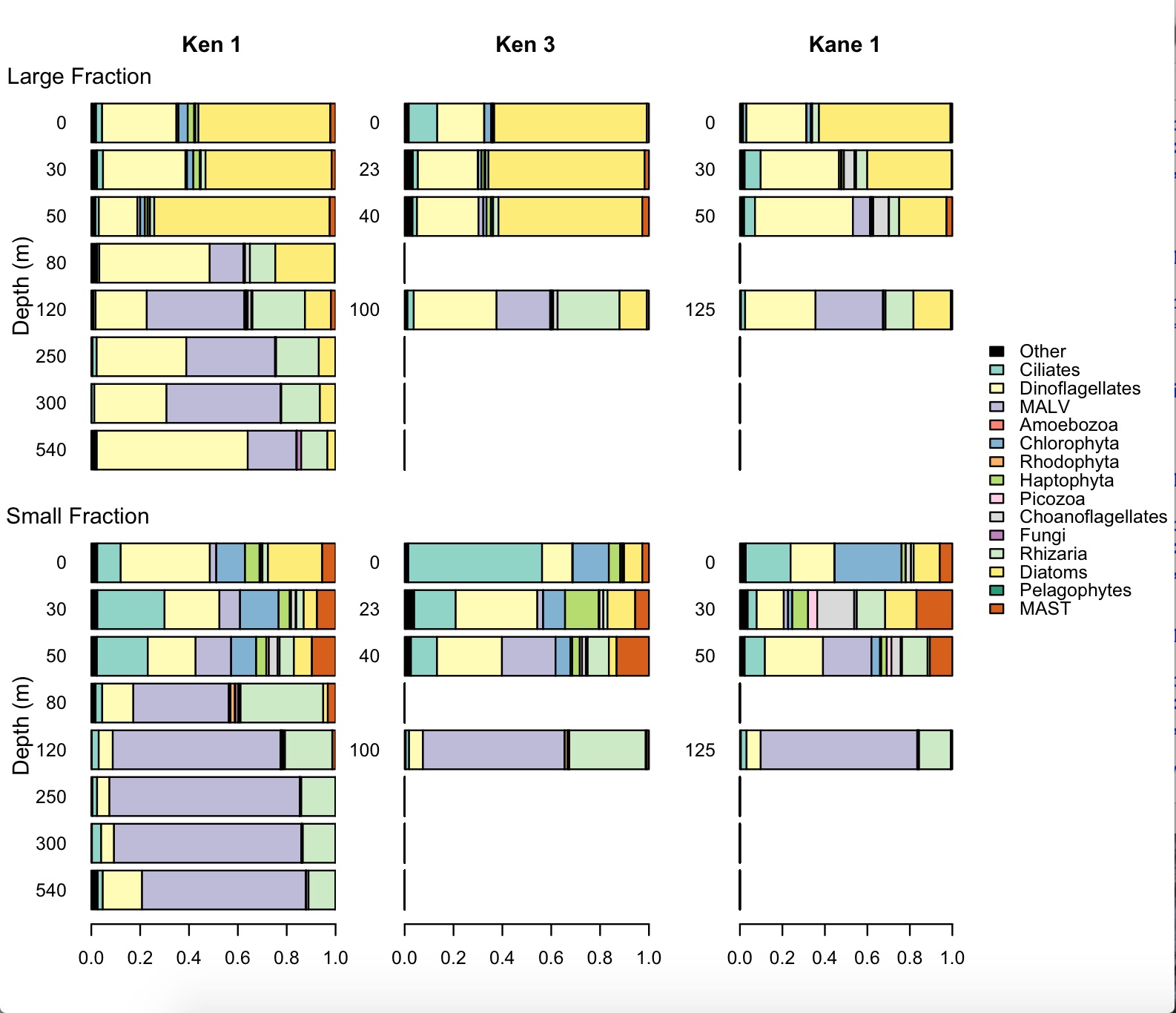
**

**Supplementary Figure S3-1.** Relative abundances of microbial eukaryotic taxa detected in Nares Strait stations: Ken1, Ken 3 and Kane 1 in 2014 from 18S rDNA amplicon sequencing.

**
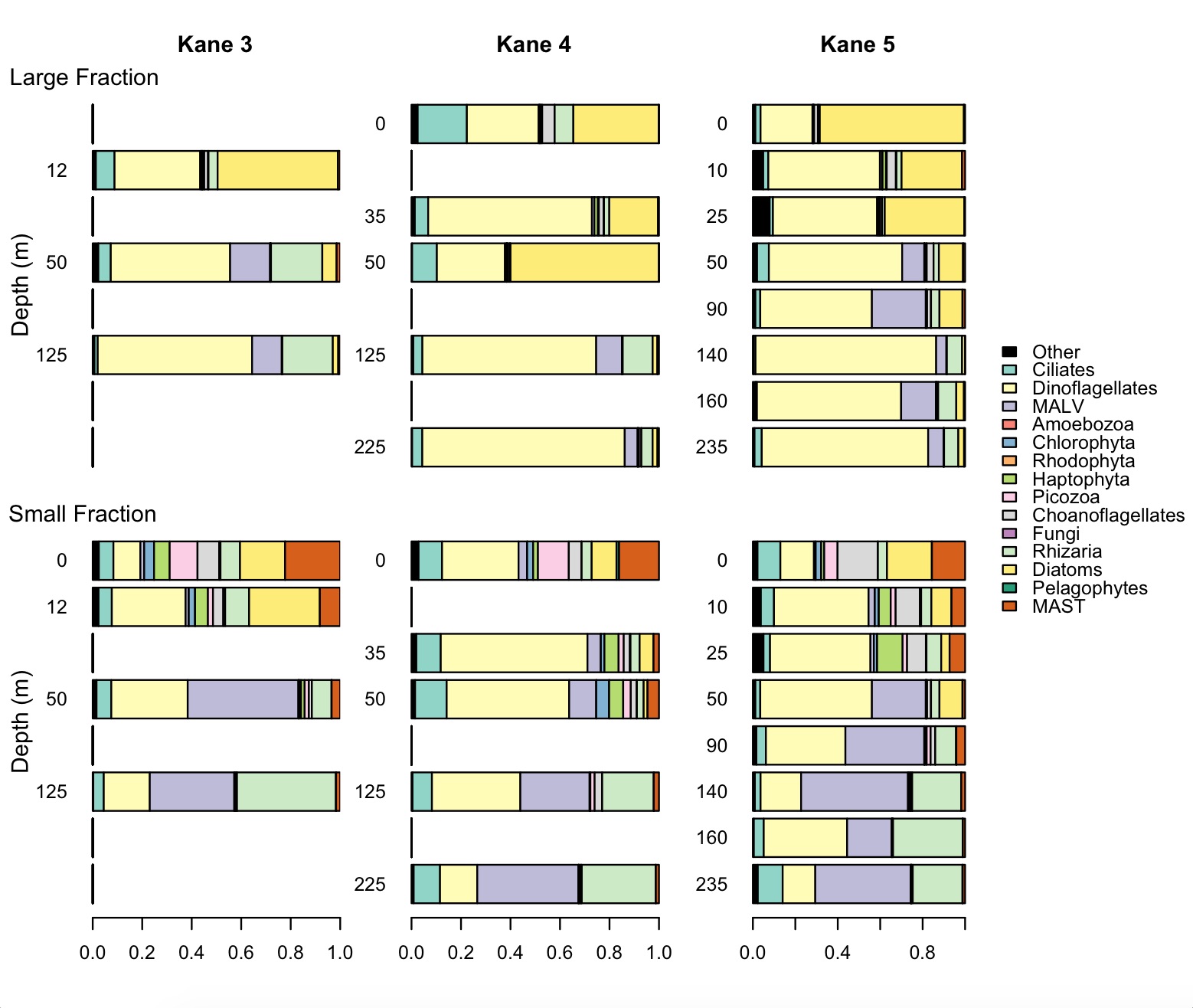
**

**Supplementary Figure S3-2.** Relative abundances of microbial eukaryotic taxa detected in Nares Strait stations: Kane 3, Kane 4, and Kane 5 in 2014 from 18S rDNA amplicon sequencing.

**
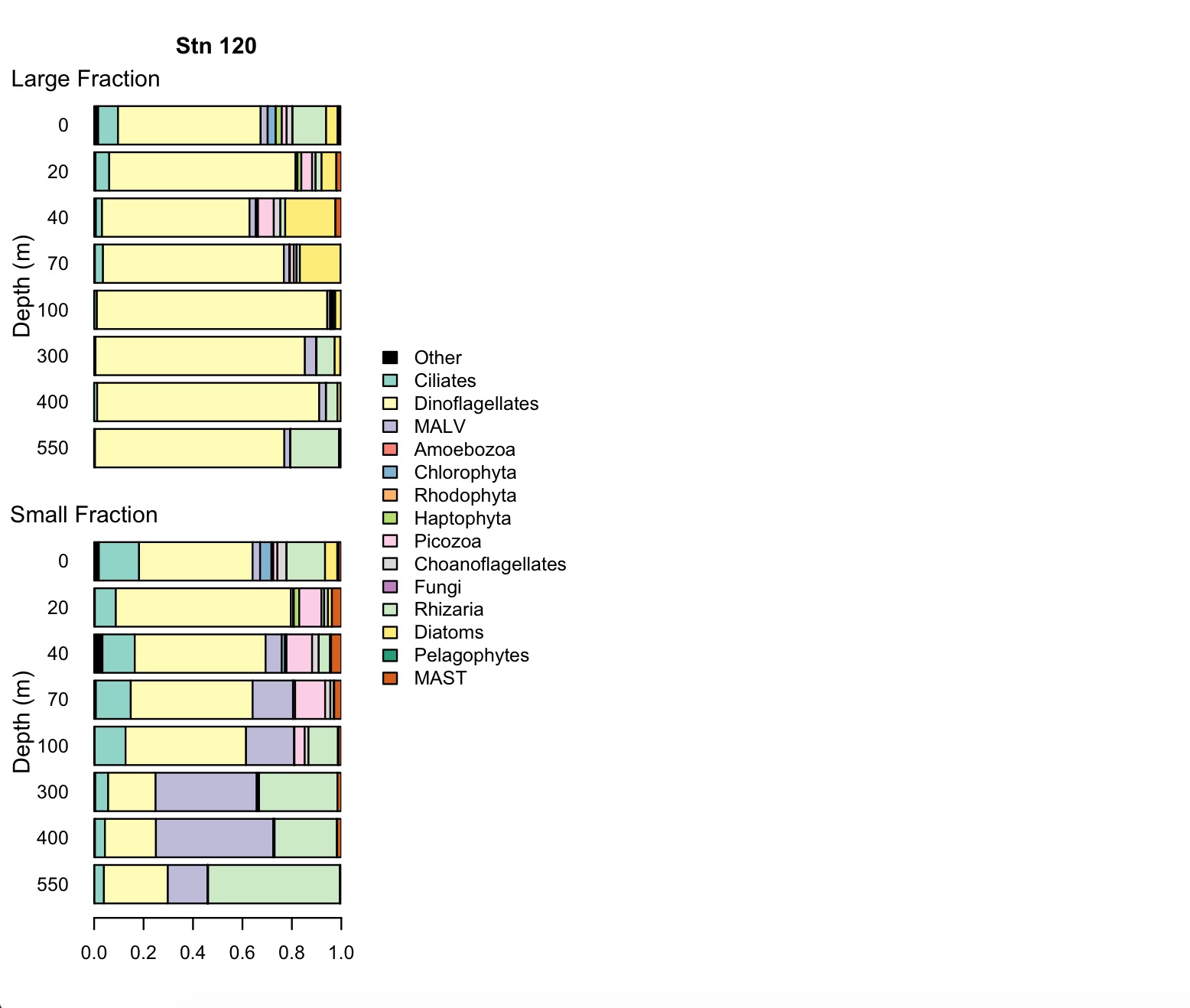
**

**Supplementary Figure S3-3.** Relative abundances of microbial eukaryotic taxa detected in below Nares Strait at station 120 in *Pikialasorsuaq* in 2014 from 18S rDNA amplicon sequencing.


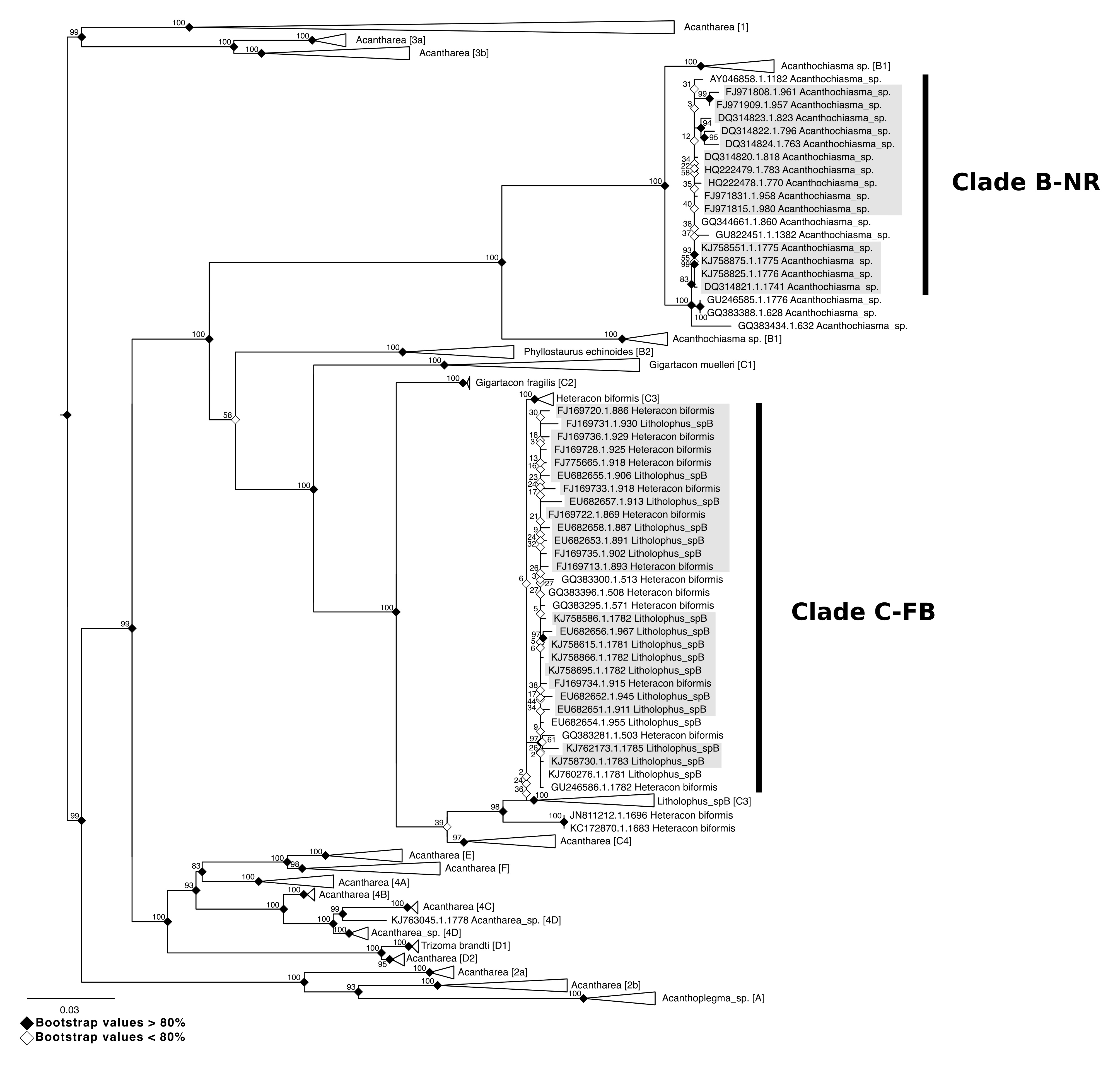
**Supplementary Figure S4.** Maximum likelihood (ML) phylogenetic tree of Acantharia inferred from 18S rRNA gene sequences from the PR2 database (Guillou *et al.* 2012) and including nearly full length 18S rDNA sequences from environmental NCBI entries. The tree was constructed using the TIM2+F+R4 substitution model in IQTREE. Percentage support from 1,000 pseudoreplicates are shown at the nodes with diamonds. Tips are labelled with NCBI GenBank accession number, taxon name, and clade in square brackets following Decelle *et al.* (2012b). Scale bar shows number of substitutions per site over a total sequence length of 1058 base pairs. Sequences from the Arctic are highlighted with grey boxes. See Supplementary Table S5 for details.

**References;**

Decelle, J., Suzuki, N., Mahé, F., de Vargas, C., and Not, F. (2012b) Molecular phylogeny and morphological evolution of the Acantharia (Radiolaria). *Protist,* **163**, 435–450. doi: 10.1016/j.protis.2011.10.002

Guillou, L., Bachar, D., Audic, S., Bass, D., Berney, C., Bittner, L., Boutte, C., Burgaud, G., et al. (2012) The Protist Ribosomal Reference database (PR2): a catalog of unicellular eukaryote Small Sub-Unit rRNA sequences with curated taxonomy. *Nucleic Acids Res*., **41**, D597–604. doi: 10.1093/nar/gks1160
