# Supplementary material for: Environmental selection and advective transport shape the distribution of two cyst-forming Acantharia clades in the Canadian Arctic
Source: J Plankton Res. 2024 Oct 4;46(6):542–54. doi: 10.1093/plankt/fbae051 (PMC11629782; doi:10.1093/plankt/fbae051)
Supplement: Supplementary_Table_S5_Thaler_fbae051 [file supplementary_table_s5_thaler_fbae051.docx]

Supplementary Table S5. Sequences from Arctic 18S rRNA gene clone libraries belonging to Acantharia clades B-NR and C-FB and used in the phylogenetic tree in Figure S4 and Figure 4. Relevant publications are given under Reference. Details for unpublished sequences were verified from field and laboratory notes kept in the Lovejoy Laboratory. Taxonomy from PR2 v4.14. Sample regions: Canada Basin (CB), Northwind Ridge (NR), Franklin Bay (FB), Northern Baffin Bay (NBB). Length of sequences in base pairs (bp), Sample (Depth) in meters,

| **GenBank** | **clade** | **genus** | **species** | **bp** | **Clone** | **Lat °N** | **Long_°W** |
| --- | --- | --- | --- | --- | --- | --- | --- |
| FJ971815 | Acantharea_B1 | *Acanthochiasma* | Acanthochiasma_sp. | 980 | Ar1353d43 | 76.999 | 140.187 |
| DQ314820 | Acantharea_B1 | *Acanthochiasma* | Acanthochiasma_sp. | 818 | NW614.35 | 75.987 | 156.869 |
| FJ971808 | Acantharea_B1 | *Acanthochiasma* | Acanthochiasma_sp. | 961 | Ar1353c14 | 76.999 | 140.187 |
| DQ314823 | Acantharea_B1 | *Acanthochiasma* | Acanthochiasma_sp. | 823 | NW614.52 | 75.987 | 156.869 |
| FJ971909 | Acantharea_B1 | *Acanthochiasma* | Acanthochiasma_sp. | 957 | Ar1663d40 | 75.845 | 140.187 |
| FJ971831 | Acantharea_B1 | *Acanthochiasma* | Acanthochiasma_sp. | 958 | Ar1663c63 | 76.999 | 140.187 |
| DQ314821 | Acantharea_B1 | *Acanthochiasma* | Acanthochiasma_sp. | 1741 | NW614.49 | 75.987 | 156.869 |
| KJ758551 | Acantharea_B1 | *Acanthochiasma* | Acanthochiasma_sp. | 1775 | SGYU1204 | 73.42 | 157.4 |
| KJ758825 | Acantharea_B1 | *Acanthochiasma* | Acanthochiasma_sp. | 1776 | SGYU444 | 73.42 | 157.4 |
| KJ758875 | Acantharea_B1 | *Acanthochiasma* | Acanthochiasma_sp. | 1775 | SGYU507 | 73.42 | 157.4 |
| DQ314822 | Acantharea_B1 | *Acanthochiasma* | Acanthochiasma_sp. | 796 | NW614.60 | 75.987 | 156.869 |
| DQ314824 | Acantharea_B1 | *Acanthochiasma* | Acanthochiasma_sp. | 763 | NW414.18 | 76.783 | 148.965 |
| HQ222479 | Acantharea_B1 | *Acanthochiasma* | Acanthochiasma_sp. | 783 | CB1901S42 | 73.98 | 140.09 |
| HQ222478 | Acantharea_B1 | *Acanthochiasma* | Acanthochiasma_sp. | 770 | CB1901S43 | 73.98 | 140.09 |
| FJ169720 | Acantharea_C3 | *Heteracon_C3* | Heteracon_C3_biformis | 886 | CS163S39 | 70.02 | 126.42 |
| FJ169736 | Acantharea_C3 | *Heteracon_C3* | Heteracon_C3_biformis | 929 | CS050L21 | 70.02 | 126.42 |
| FJ169722 | Acantharea_C3 | *Heteracon_C3* | Heteracon_C3_biformis | 869 | CS163S48 | 70.02 | 126.42 |
| FJ775665 | Acantharea_C3 | *Heteracon_C3* | Heteracon_C3_biformis | 918 | CS060S27 | 70.02 | 126.42 |
| FJ169728 | Acantharea_C3 | *Heteracon_C3* | Heteracon_C3_biformis | 925 | CS123L18 | 70.02 | 126.42 |
| FJ169734 | Acantharea_C3 | *Heteracon_C3* | Heteracon_C3_biformis | 915 | CS050L17 | 70.02 | 126.42 |
| FJ169733 | Acantharea_C3 | *Heteracon_C3* | Heteracon_C3_biformis | 918 | CS050S32 | 70.02 | 126.42 |
| FJ169713 | Acantharea_C3 | *Heteracon_C3* | Heteracon_C3_biformis | 893 | CS060L18 | 70.02 | 126.42 |
| FJ169731 | Acantharea_C3 | *Litholophus* | Litholophus_spB | 930 | CS050S22 | 70.02 | 126.42 |
| EU682657 | Acantharea_C3 | *Litholophus* | Litholophus_spB | 913 | 05M100r.02 | 71.561 | 140.112 |
| EU682653 | Acantharea_C3 | *Litholophus* | Litholophus_spB | 891 | 05M100n.13 | 71.561 | 140.112 |
| EU682651 | Acantharea_C3 | *Litholophus* | Litholophus_spB | 911 | 05M100n.01 | 71.561 | 140.112 |
| FJ169735 | Acantharea_C3 | *Litholophus* | Litholophus_spB | 902 | CS050L19 | 70.02 | 126.42 |
| EU682658 | Acantharea_C3 | *Litholophus* | Litholophus_spB | 887 | 05M100r.14 | 71.561 | 140.112 |
| EU682655 | Acantharea_C3 | *Litholophus* | Litholophus_spB | 906 | 05M100n.33 | 71.561 | 140.112 |
| EU682656 | Acantharea_C3 | *Litholophus* | Litholophus_spB | 967 | 05M100n.36 | 71.561 | 140.112 |
| EU682652 | Acantharea_C3 | *Litholophus* | Litholophus_spB | 945 | 05M100n.05 | 71.561 | 140.112 |
| KJ758586 | Acantharea_C3 | *Litholophus* | Litholophus_spB | 1782 | SGYU1251 | 73.42 | 157.4 |
| KJ758615 | Acantharea_C3 | *Litholophus* | Litholophus_spB | 1781 | SGYU1291 | 73.42 | 157.4 |
| KJ758695 | Acantharea_C3 | *Litholophus* | Litholophus_spB | 1782 | SGYU1409 | 73.42 | 157.4 |
| KJ758730 | Acantharea_C3 | *Litholophus* | Litholophus_spB | 1783 | SGYU1464 | 73.42 | 157.4 |
| KJ758866 | Acantharea_C3 | *Litholophus* | Litholophus_spB | 1782 | SGYU495 | 73.42 | 157.4 |
| KJ762173 | Acantharea_C3 | *Litholophus* | Litholophus_spB | 1785 | SGYT576 | 73.42 | 157.4 |
| EU182807 | Acantharea_C3 | *Litholophus* | Litholophus_spA | 493 | A047DL17 | 77.335 | 76.021 |

Region, Depth and reference for the GenBank sequences.

| **GenBank** | **Region** | **depth** | **sample_date** | **Reference** |
| --- | --- | --- | --- | --- |
| FJ971815 | CB | 57 | 15-Aug-2007 | Scarcella et al. unpublished |
| DQ314820 | NR | 50 | 28-30 August 2002 | Lovejoy et al. 2006 |
| FJ971808 | CB | 57 | 15-Aug-2007 | Scarcella et al. unpublished |
| DQ314823 | NR | 50 | 28-30 August 2002 | Lovejoy et al. 2006 |
| FJ971909 | CB | 70 | 20-Aug-2007 | Scarcella et al. unpublished |
| FJ971831 | CB | 70 | 20-Aug-2007 | Scarcella et al. unpublished |
| DQ314821 | NR | 50 | 28-30 August 2002 | Lovejoy et al. 2006 |
| KJ758551 | NR | 500 | 11-Aug-2002 | Lie et al. 2014 |
| KJ758825 | NR | 500 | 11-Aug-2002 | Lie et al. 2014 |
| KJ758875 | NR | 500 | 11-Aug-2002 | Lie et al. 2014 |
| DQ314822 | NR | 50 | 28-30 August 2002 | Lovejoy et al. 2006 |
| DQ314824 | NR | 50 | 28-30 August 2002 | Lovejoy et al. 2006 |
| HQ222479 | CB | 70 | 21-Aug-2007 | Scarcella et al. unpublished |
| HQ222478 | CB | 70 | 21-Aug-2007 | Scarcella et al. unpublished |
| FJ169720 | FB | 220 | 16-juil-04 | Terrado et al. 2009 |
| FJ169736 | FB | 161 | 19-nov-03 | Terrado et al. 2009 |
| FJ169722 | FB | 220 | 16-juil-04 | Terrado et al. 2009 |
| FJ775665 | FB | 219 | 22-Dec-2003 | Terrado et al. 2009 |
| FJ169728 | FB | 225 | 22-Apr-2004 | Terrado et al. 2009 |
| FJ169734 | FB | 161 | 19-nov-03 | Terrado et al. 2009 |
| FJ169733 | FB | 161 | 19-nov-03 | Terrado et al. 2009 |
| FJ169713 | FB | 219 | 22-Dec-2003 | Terrado et al. 2009 |
| FJ169731 | FB | 161 | 19-nov-03 | Terrado et al. 2009 |
| EU682657 | CB | 30 | 05-sept-05 | Lovejoy & Potvin 2010 |
| EU682653 | CB | 30 | 05-sept-05 | Lovejoy & Potvin 2010 |
| EU682651 | CB | 30 | 05-sept-05 | Lovejoy & Potvin 2010 |
| FJ169735 | FB | 161 | 19-nov-03 | Terrado et al. 2009 |
| EU682658 | CB | 30 | 05-sept-05 | Lovejoy & Potvin 2010 |
| EU682655 | CB | 30 | 05-sept-05 | Lovejoy & Potvin 2010 |
| EU682656 | CB | 30 | 05-sept-05 | Lovejoy & Potvin 2010 |
| EU682652 | CB | 30 | 05-sept-05 | Lovejoy & Potvin 2010 |
| KJ758586 | NR | 500 | 11-Aug-2002 | Lie et al. 2014 |
| KJ758615 | NR | 500 | 11-Aug-2002 | Lie et al. 2014 |
| KJ758695 | NR | 500 | 11-Aug-2002 | Lie et al. 2014 |
| KJ758730 | NR | 500 | 11-Aug-2002 | Lie et al. 2014 |
| KJ758866 | NR | 500 | 11-Aug-2002 | Lie et al. 2014 |
| KJ762173 | NR | 35 | 12-Aug-2002 | Lie et al. 2014 |
| EU182807 | NBB | 540 | 21-Aug-2005 | Hamilton et al. 2008 |

Full References:

| Li, W. K. W., Andersen, R. A., Gifford, D. J., Incze, L. S., Martin, J. L., Pilskaln, C. H., Rooney-Varga, J. N., Sieracki, M. E., et al. (2011). Planktonic microbes in the Gulf of Maine Area. PLoS One, 6, e20981. doi: 10.1371/journal.pone.0020981 |
| --- |
| Lovejoy, C., Massana, R., and Pedrós-Alió, C. (2006) Diversity and distribution of marine microbial eukaryotes in the Arctic Ocean and adjacent seas. Appl. Environ. Microbiol., 72, 3085–3095. doi: 10.1128/AEM.72.5.3085-3095.2006 |
| Lovejoy, C., and Potvin, M. (2010) Microbial eukaryotic distribution in a dynamic Beaufort Sea and the Arctic Ocean. J. Plankt. Res., 33, 431–444. doi: 10.1093/plankt/fbq124 |
| Terrado, R., Vincent, W. F., and Lovejoy, C. (2009) Mesopelagic protists: diversity and succession in a coastal Arctic ecosystem. Aquat. Microb. Ecol., 56, 25–40. doi: 10.3354/ame01327 |
| Scarcella, K, Terrado, R, Lovejoy, C. Canada Basin microbial eukaryotes contribution to the Interaltional Polar Year. Unpublished. |
| Additional information on the Scarcella data:  Scarcella, Karen (2009): La diversité des communautés microbiennes eucaryotes actives dans les océans canadiens : analyses moléculaires de la diversité du gène d'ARNr 18S et de la nitrate réductase assimilatrice. MSc Mémoire, Département de biologie, Université Laval, Québec http://hdl.handle.net/20.500.11794/20913 |
